# Drug prescription patterns and their association with mortality and hospitalization duration in COVID-19 patients: insights from big data

**DOI:** 10.3389/fpubh.2023.1280434

**Published:** 2023-12-18

**Authors:** Reza Mehrizi, Ali Golestani, Mohammad-Reza Malekpour, Hossein Karami, Mohammad Mahdi Nasehi, Mohammad Effatpanah, Hossein Ranjbaran, Zahra Shahali, Ali Akbari Sari, Rajabali Daroudi

**Affiliations:** ^1^National Center for Health Insurance Research, Tehran, Iran; ^2^Non-Communicable Diseases Research Center, Endocrinology and Metabolism Population Sciences Institute, Tehran University of Medical Sciences, Tehran, Iran; ^3^Pediatric Neurology Research Center, Research Institute for Children Health, Shahid Beheshti University of Medical Sciences, Tehran, Iran; ^4^School of Medicine, Imam Khomeini Hospital, Tehran University of Medical Sciences, Tehran, Iran; ^5^Immunogenetics Research Center, Faculty of Medicine, Mazandaran University of Medical Sciences, Sari, Iran; ^6^Department of Health Management, Policy and Economics, School of Public Health, Tehran University of Medical Sciences, Tehran, Iran

**Keywords:** COVID-19, drug prescriptions, electronic health records, claims data, mortality

## Abstract

**Background:**

Different medication prescription patterns have been associated with varying course of disease and outcomes in COVID-19. Health claims data is a rich source of information on disease treatment and outcomes. We aimed to investigate drug prescription patterns and their association with mortality and hospitalization via insurance data for a relatively long period of the pandemic in Iran.

**Methods:**

We retrieved hospitalized patients’ data from Iran Health Insurance Organization (IHIO) spanning 26 months (2020–2022) nationwide. Included were patients with ICD-10 codes U07.1/U07.2 for confirmed/suspected COVID-19. A case was defined as a single hospitalization event for an individual patient. Multiple hospitalizations of a patient within a 30-day interval were aggregated into a single case, while hospitalizations with intervals exceeding 30 days were treated as independent cases. The Anatomical Therapeutic Chemical (ATC) was used for medications classification. The two main study outcomes were general and intensive care unit (ICU) hospitalization periods and mortality. Besides, various demographic and clinical associate factors were analyzed to derive the associations with medication prescription patterns and study outcomes using accelerated failure time (AFT) and logistic regression models.

**Results:**

During the 26 months of the study period, 1,113,678 admissions with COVID-19 diagnosis at hospitals working in company with IHIO were recorded. 917,198 cases were detected from the database, among which 51.91% were females and 48.09% were males. Among the main groups of medications, antithrombotics (55.84% [95% CI: 55.74–55.94]), corticosteroids (54.14% [54.04–54.24]), and antibiotics (42.22% [42.12–42.32]) were the top used medications among cases with COVID-19. Investigation of the duration of hospitalization based on main medication groups showed antithrombotics (adjusted median ratio = 0.94 [0.94–0.95]) were significantly associated with shorter periods of overall hospitalization. Also, antithrombotics (adjusted odds ratio = 0.74 [95%CI, 0.73–0.76]), corticosteroids (0.97 [0.95–0.99]), antivirals (0.82 [0.80–0.83]), and ACE inhibitor/ARB (0.79 [0.77–0.80]) were significantly associated with lower mortality.

**Conclusion:**

Over 2 years of investigation, antithrombotics, corticosteroids, and antibiotics were the top medications for hospitalized patients with COVID-19. Trends in medication prescription varied based on various factors across the country. Medication prescriptions could potentially significantly impact the trends of mortality and hospitalization during epidemics, thereby affecting both health and economic burdens.

## 1 Introduction

Although the COVID-19 pandemic is no longer a public health emergency of international concern, as recently stated by the World Health Organization (WHO) Director-General (1), its vast impacts on human life and society are remarkable, and many hidden aspects of this disease still need investigation. At this time, an ongoing effort to know such pandemics and gain preparedness for future similar public health events is required and highly suggested (2). Iran was one of the countries facing many challenges handling this pandemic with several waves of the disease while struggling with a high burden of non-communicable diseases (NCDs) and health system shortages that brought up a specific situation (3–5). One of the cornerstones of management of this pandemic was the way of handling severe cases admitted to hospitals and the guidelines used to treat patients effectively and in a timely manner to prevent adverse outcomes of this disease (6–8).

Health claims data is a rich source of information on the diagnosis of different diseases, pharmacological and non-pharmacological treatments for diseases, out-patient and in-patient visits, and other associated data which is used by health systems and insurance companies to track the overall performance of the health authorities and it has shown that its data is a valuable tool to measure the quality of provided care (9, 10). While previous ecological studies used medication sales data to estimate drug prescription patterns and their adherence to clinical guidelines, using health claims data as individual-based database, could offer a deeper understanding of evidence-based treatment and its changes over time (11, 12). In the case of COVID-19, the claims data proved effective in drawing different aspects of disease management and its impact on patient outcomes (13, 14). Research in this field shows that the prescription of different medications could majorly affect the course of disease and outcomes like mortality in patients with COVID-19 infection (14, 15). Previous research on this topic showed that the most used medications in hospitalized patients with suspected or confirmed COVID-19 were corticosteroids, antithrombotics, and antibiotics, each varying impact on infection course and outcomes (16, 17).

The Iran Health Insurance Organization (IHIO) is one of the most prominent governmental insurance companies, providing coverage for more than 40 million Iranians, which offers basic insurance coverage that is also affordable (18, 19). IHIO, in the past years of its activity, has developed a rich source of claims data that could be used to assess the performance of health system and physicians on a large scale (14). In this study, we aimed to investigate the claims data from IHIO on drug prescription patterns and their association with mortality and hospitalization duration in COVID-19 patients to draw the associations between utilization of various medications and COVID-19 disease course and outcomes. Health authorities could use this study’s findings to refine the prescription of medications for similar conditions of public health emergencies like COVID-19 in the future.

## 2 Materials and methods

### 2.1 Study design and population

This retrospective study recruited the IHIO data from hospitals collaborating with this insurance organization in all provinces of Iran on patients admitted with COVID-19 for the February 1, 2020-March 20, 2022 period. About 42 million residents of Iran were under coverage of IHIO during the period of study (19). Patients admitted and registered with International Classification of Diseases 10^th^ revision (ICD-10) codes U07.1 (COVID-19 diagnosis by serological tests) and U07.2 (COVID-19 diagnosis by a physician based on clinical signs and symptoms) (20) were extracted from the IHIO hospitalization database for this study, resulted in totally 1,113,678 admissions. The retrieved data included demographic, hospitalization, and medication information of the included population. Access to the fully anonymized data was granted to researchers in January 2023, at which point they commenced their analysis.

### 2.2 Data source variables

In the retrieved hospitalization dataset, numerous variables pertained to each admission. Each patient was assigned a unique patient code, which allowed for the identification of multiple admissions by the same patient. COVID-19 diagnoses, coded as U07.1 and U07.2 according to ICD-10, were present in the dataset. Demographic characteristics of patients, including ages ranging from 1 to 105 years, and gender (male/female), were also included. The IHIO comprises various main funds, such as Civil Servants, Rural, Iranian, Universal Health Insurance, Foreign Citizens, and Other Social Strata funds. The assignment to a particular fund is determined by factors such as occupation, income, and socioeconomic status (21).

The dataset also included the province of hospitalization, covering all provinces in Iran. It noted the type of admission, distinguishing between ward and emergency department admissions, which may relate to the patient’s condition upon initial hospital arrival, with more severe cases often directly admitted to wards when beds are available. The number of total hospitalization days, including days spent in the intensive care unit (ICU), was recorded, and any instances of more than 90 days were considered outliers and deemed inappropriate for analysis. Dates of admission and discharge were also part of the dataset. The admission date was categorized by the month of hospitalization, which, considering different COVID-19 variants and vaccine accessibility during the pandemic, may be relevant to the disease’s severity.

Physician specialties responsible for managing patients were classified into main groups, including General Practitioners (GPs), infectious diseases specialists, internal medicine specialists (all internal specialties excluding pulmonologists), emergency physicians, pediatricians, cardiologists, pulmonologists, others, and unknown. The categorization considered that more complex and serious cases would typically be managed by specialists, potentially relating to the severity of the disease. The dataset further encompassed medications prescribed during hospitalization, either in brand or generic names. Furthermore, the hospitalization outcome was recorded, distinguishing between cases of recovery and mortality.

### 2.3 Medication classification

Medications assessed in this study were classified based on the Anatomical Therapeutic Chemical (ATC) Classification developed by the WHO (22). The ATC classification divides medications according to their therapeutic, pharmacological, and chemical properties in groups at five levels, including (#1) fourteen main anatomical or pharmacological groups of medications named A to V, (#2) pharmacological or therapeutic subgroup, (#3 and #4) chemical, pharmacological or therapeutic subgroup, and (#5) chemical substance (22). Our dataset lacked ATC codes for the prescribed medications, providing only generic or brand names. This led to the possibility of a single medication having multiple names in the dataset. To address this issue and standardize the ATC codes for different brand and generic names of medications, we followed a multi-step process.

First, we compiled a comprehensive list of all prescribed medications from our dataset. Next, we merged the string name of this list medications with a dataset containing generic drug names along with their associated ATC codes, which obtained from the Iran Food and Drug Administration website (23). For medications where ATC codes could not be automatically determined through this process, we manually inputted the codes. Subsequently, we utilized the first two levels of ATC codes to classify medications into major groups. For example, medications falling under the B01 code were classified as antithrombotic agents, while those under H02 were categorized as corticosteroids. The ATC code of included medications are presented in Table 1.

**Table 1:** Frequency of medication prescriptions among cases with COVID-19 in different groups of drugs during the study period.

**Medication groups**	**ATC code**	**Frequency, No. (%; 95% CI)**
**Antithrombotics**		512,191 (55.84%; 55.74–55.94)
Heparin	B01AB01	386,626 (42.15%; 42.05–42.25)
Enoxaparin	B01AB05	178,376 (19.45%; 19.37–19.53)
Aspirin	B01AC06	175,210 (19.1%; 19.02–19.18)
Clopidogrel	B01AC04	32,485 (3.54%; 3.50–3.58)
Warfarin	B01AA03	5,518 (0.6%; 0.59–0.62)
**Corticosteroids**		496,547 (54.14%; 54.04–54.24)
Dexamethasone	H02AB02	435,233 (47.45%; 47.35–47.55)
Methylprednisolone	H02AB04	96,275 (10.5%; 10.43–10.56)
Hydrocortisone	H02AB09	62,029 (6.76%; 6.71–6.81)
Prednisolone	H02AB06	27,204 (2.97%; 2.93–3.00)
Betamethasone (IM/IV)	H02AB01	5,216 (0.57%; 0.55–0.58)
**Antibiotics**		387,251 (42.22%; 42.12–42.32)
Ceftriaxone	J01DD04	265,519 (28.95%; 28.86–29.04)
Azithromycin	J01FA10	82,908 (9.04%; 8.98–9.10)
Vancomycin	J01XA01	87,093 (9.5%; 9.44–9.56)
Meropenem	J01DH02	78,881 (8.6%; 8.54–8.66)
Cefepime	J01DE01	36,435 (3.97%; 3.93–4.01)
Ceftazidime	J01DD02	36,887 (4.02%; 3.98–4.06)
Imipenem	J01DH51	27,703 (3.02%; 2.99–3.06)
Cefotaxime	J01DD01	7,409 (0.81%; 0.79–0.83)
Levofloxacin	J01MA12	4,955 (0.54%; 0.53–0.56)
Ceftizoxime	J01DD07	3,813 (0.42%; 0.40–0.43)
**Gastrointestinal drugs**		381,269 (41.57%; 41.47–41.67)
Pantoprazole	A02BC02	254,333 (27.73%; 27.64–27.82)
Famotidine	A02BA03	183,064 (19.96%; 19.88–20.04)
Omeprazole	A02BC01	4,372 (0.48%; 0.46–0.49)
**Antivirals**		366,763 (39.99%; 39.89–40.09)
Remdesivir	J05AB16	339,539 (37.02%; 36.92–37.12)
Lopinavir/ritonavir	J05AR10	17,291 (1.89%; 1.86–1.91)
Favipiravir	J05AX27	13,982 (1.52%; 1.50–1.55)
Acyclovir	J05AB01	5,292 (0.58%; 0.56–0.59)
**ACE inhibitor/ARB**		118,385 (12.91%; 12.84–12.98)
Losartan	C09CA01	82,608 (9.01%; 8.95–9.07)
Captopril	C09AA01	33,488 (3.65%; 3.61–3.69)
Valsartan	C09CA03	14,288 (1.56%; 1.53–1.58)
**Diuretics**		106,950 (11.66%; 11.59–11.73)
Furosemide	C03CA01	97,074 (10.58%; 10.52–10.65)
Spironolactone	C03DA01	20,886 (2.28%; 2.25–2.31)
Hydrochlorothiazide	C03AA03	6,811 (0.74%; 0.73–0.76)
**Antidiabetics**		106,120 (11.57%; 11.50–11.64)
Insulins	A10AB01 A10AE01 A10AB05 A10AB30 A10AB06 A10AE05 A10AB04	93,332 (10.18%; 10.11–10.24)
Metformin	A10BA02	22,247 (2.43%; 2.39–2.46)
**Inhalants**		96,169 (10.49%; 10.42–10.55)
Fluticasone/salmeterol	R03AK06	78,168 (8.52%; 8.47–8.58)
Salbutamol	R03CC02	57,786 (6.3%; 6.25–6.35)
Atrovent	R03BB01	30,356 (3.31%; 3.27–3.35)
**Immunostimulants**		66,599 (7.26%; 7.21–7.31)
Interferon beta-1a	L03AB07	59,932 (6.53%; 6.48–6.58)
Interferon beta-1b	L03AB08	4,890 (0.53%; 0.52–0.55)
Filgrastim (GCSF)	L03AA02	2,657 (0.29%; 0.28–0.30)
**Others**		-
Atorvastatin	C10AA05	200,378 (21.85%; 21.76–21.93)
Nystatin	A07AA02	19,659 (2.14%; 2.11–2.17)
Amlodipine	C08CA01	47,079 (5.13%; 5.09–5.18)
Metoprolol	C07AB02	39,836 (4.34%; 4.30–4.38)
Nitroglycerin	C01DA02	63,100 (6.88%; 6.83–6.93)
Norepinephrine	C01CA03	32,113 (3.5%; 3.46–3.54)
Digoxin	C01AA05	14,729 (1.61%; 1.58–1.63)
Vitamin D3	A11CC05	98,154 (10.7%; 10.64–10.76)
Vitamin C	A11GA01	12,351 (1.35%; 1.32–1.37)
Hydroxychloroquine	P01BA02	25,818 (2.81%; 2.78–2.85)
Colchicine	M04AC01	23,076 (2.52%; 2.48–2.55)

### 2.4 Data preparation

Data curation and preparation in this study had two main steps to resolve the two challenges of the prescribed medications and the number of patients based on different hospitalization sessions. Regarding the first challenge, after applying the process discussed in previous section, about 6,000 medications were identified through the dataset, and their attributed ATC codes were determined. Medications routinely used during hospitalization (e.g., serums and solutions) were excluded from the list of medications finalized for analysis. Medications were included if they met either criteria: a prescription frequency of more than 0.05% among the hospitalized cases or proven effectiveness on COVID-19 based on guidelines or previous evidence. We assumed medications which were prescribed in less than 0.05% of cases and were not mentioned in guidelines were unimportant rare prescribed medications and including them in a big data analysis with nearly 1 million cases would not add any more significance to our analysis. This approach reduced the number of medications to 52, and the final list of the major groups of medications, based on the first two levels of ATC codes, included antithrombotics, antibiotics, corticosteroids, gastrointestinal (GI) medications, antivirals, inhalers, diuretics, anti-diabetics, immunostimulants, Angiotensin-converting enzyme (ACE) inhibitors and angiotensin II receptor blockers (ARBs), and other miscellaneous drugs.

The second main step of data preparation was to define cases in this study. During the initial data exploration, we identified some patients who had experienced more than one hospitalization within the study period. While it is possible for individuals to infected by COVID-19 multiple times, especially given the long period of our study, a common pattern we observed involved consecutive admissions occurring within days of each other or with very short intervals. This observation can be attributed to some hospitalizations being of short duration, primarily intended for medication administration, or involving early discharges before full recovery. As a result, we aggregated all hospitalizations of a patient occurring within intervals of 30 days or less into a single hospitalization, defining it as a case that represented a single entry in the curated dataset. The date of the first hospitalization was considered for this newly defined case. Detailed information regarding changes to other variables for these cases is discussed in the Supplementary Appendix. For patients with only one admission, that admission was directly considered as a case. In instances where patients had multiple admissions with intervals exceeding 1 month, each hospitalization was treated as an independent case. This data preparation step resulted in a minor reduction in dataset size, facilitating a more rigorous analysis of the prescribed medications.

### 2.5 Statistical analysis

Following data extraction from the IHIO database, data curation, and preparation were done using Python programming software v3.10.9 by Pandas and Numpy libraries.^1^ Visualizations were done by the Matplotlib library in Python and the ggplot2 library in R programming software v4.3.1.^2^ Statistical analyses were done by Statsmodels and Lifelines libraries in Python. Quantitative variables were summarized by the mean and standard deviation (±SD) or median and interquartile range (IQR), while qualitative variables were presented in terms of frequency and percentages, and 95% confidence interval (CI) was reported for statistical significance comparison and representing effect size To investigate the contribution of different medication groups to the mortality of cases, we used univariable and multiple logistic regression, and results were reported as crude and adjusted odds ratio (OR) with an associated 95% confidence interval (CI). Regarding hospitalization duration, we treated it as a survival analysis in which recovered and deceased cases were considered events and censored for the model. Among the available options for survival analysis, we selected the accelerated failure time (AFT) model, a parametric survival model. After evaluating various distributions, we found that the log-logistic distribution provided the best fit, due to its lowest Akaike information criterion (AIC), and used it to model the outcome with different variables. The results of univariable and multiple analysis were reported as crude and adjusted median ratios (MRs), with 95% CI. Models were applied to both the all hospitalized population and patients who experienced the ICU. This approach aimed to generalize the results across both patient groups. In adjusted models for both outcomes, each drug was adjusted with sex and age as basic characteristics of cases, province and insurance fund as factors related to the socioeconomic situation of the cases, month of admission, admission type, and physician specialists as factors attributed to the severity of disease, and other drugs as a proxy of both severity of disease and the concurrent other chronic diseases, as well as considering the effect of treatment with multiple medications. Two-tailed *p*-values<0.05 were set as the level of statistical significance for conducted analyses.

### 2.6 Ethical considerations

This study was done according to the Declaration of Helsinki guidelines, and the study protocol was reviewed by the ethical committee at Tehran University of Medical Sciences and received ethical approval before initiation of the investigation (code: IR.TUMS.SPH.REC.1401.120). Considering this study was a retrospective study on medical records, The provided data by IHIO in this study were fully anonymized before investigators had access to it, and the requirement for informed consent was not necessary and has been waived by the ethics committee.

## 3 Results

### 3.1 General findings

During the 26 months of the study period, a total number of 1,113,678 admissions with COVID-19 diagnosis at hospitals working in company with IHIO were recorded, including 58.30% (95% CI: 58.21–58.39) with U07.1 and 41.70% (41.61–41.79) with U07.2 diagnostic code. A majority of the admissions were ward admissions (73.86% [95% CI: 73.77–73.94]) compared to the emergency department (26.14% [26.06–26.23]). General practitioners (43.63% [43.54–43.72]), internal medicine specialists (17.43% [17.36–17.50]), and infectious diseases specialists (16.82% [16.75–16.89]) were the top physician specialists managing the admissions. According to the case definition in this study, a total of 917,198 cases were detected from the database, among which 51.91% (51.81–52.01) were females and 48.09% (47.99–48.19) were males. For each sex, the 61–70 age group had the highest number of cases (total: 19.04% [18.96-19.12] (females: 19.65% [19.53-19.76], males: 18.37% [18.26-18.49])), and the 11–20 age group had the least number of cases (total: 1.78% [1.75-1.81] (females: 1.85% [1.82-1.88], males: 1.70% [1.66-1.74])).

The mortality rate among all cases was 10.36% (10.29–10.42). Among the 148,171 cases that required ICU care during their hospitalization, the mortality rate was significantly higher at 40.37% (40.13–40.62), compared to a lower mortality rate of 4.9% (4.85–4.96) in cases that did not require ICU care during their hospitalizations. The total hospitalization days [mean (95% CI): 5.78 (5.77–5.80), median (IQR): 4 (3–7)] was significantly shorter than ICU hospitalization days [mean (95% CI): 6.85 (6.81–6.89), median (IQR): 5 (2–8)].

### 3.2 Drug prescription patterns

**Figure 1:**
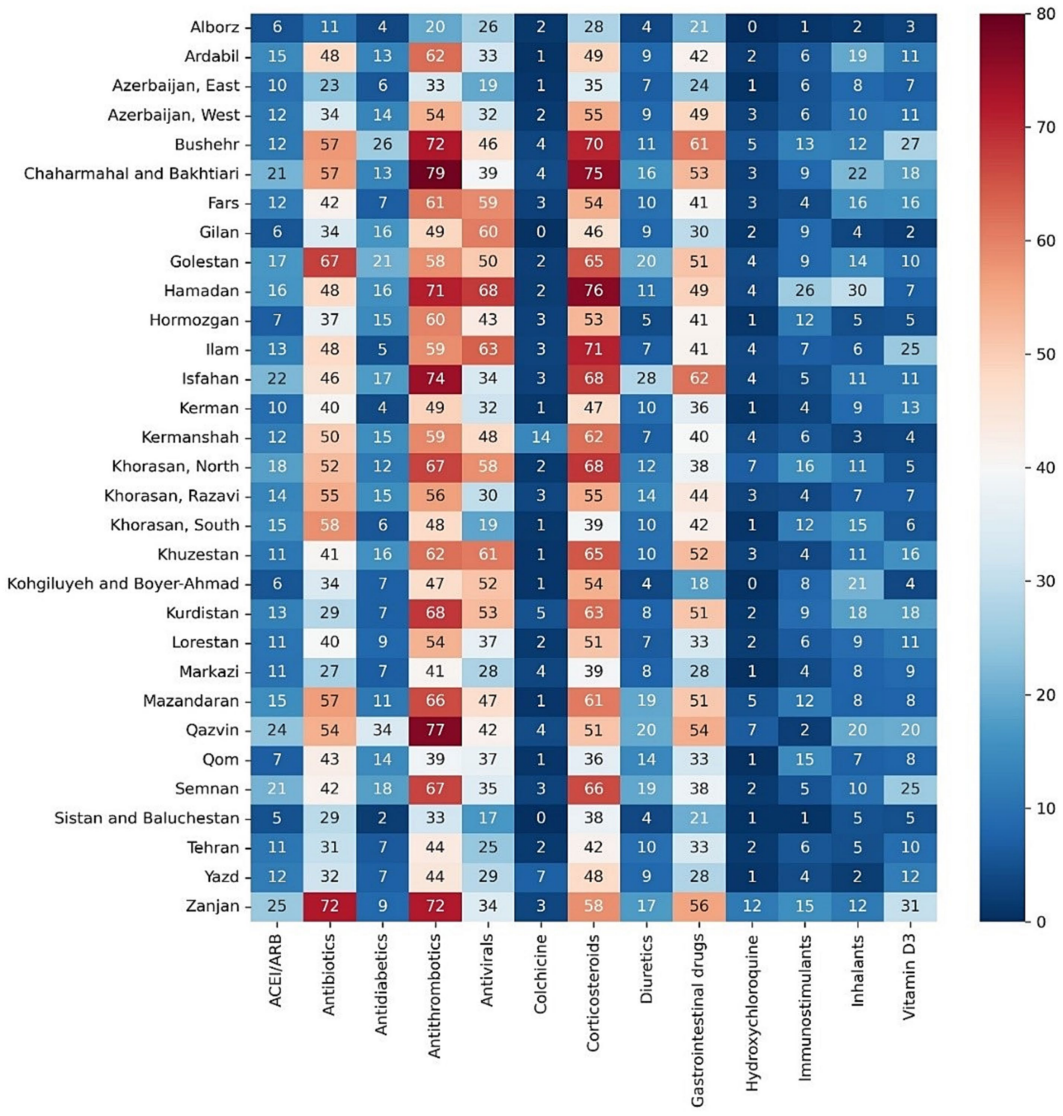
Percentage of medication prescriptions among cases with COVID-19 for different main groups of drugs in provinces of Iran.

**Figure 2:**
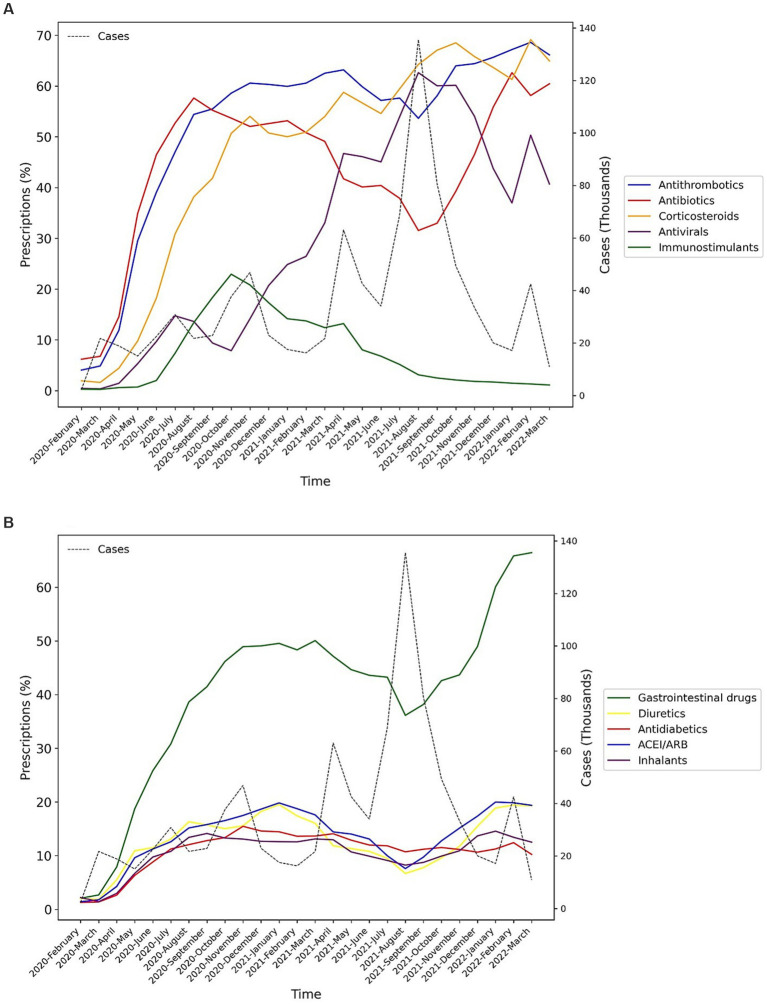
Percentage of medication prescriptions trends of the main groups of drugs during the study period (Lines in dots is the trend of the number of hospitalized COVID-19 cases). **(A)** Main drug classes in COVID-19 treatment, **(B)** Other drug classes in COVID-19 treatment.

**Figure 3:**
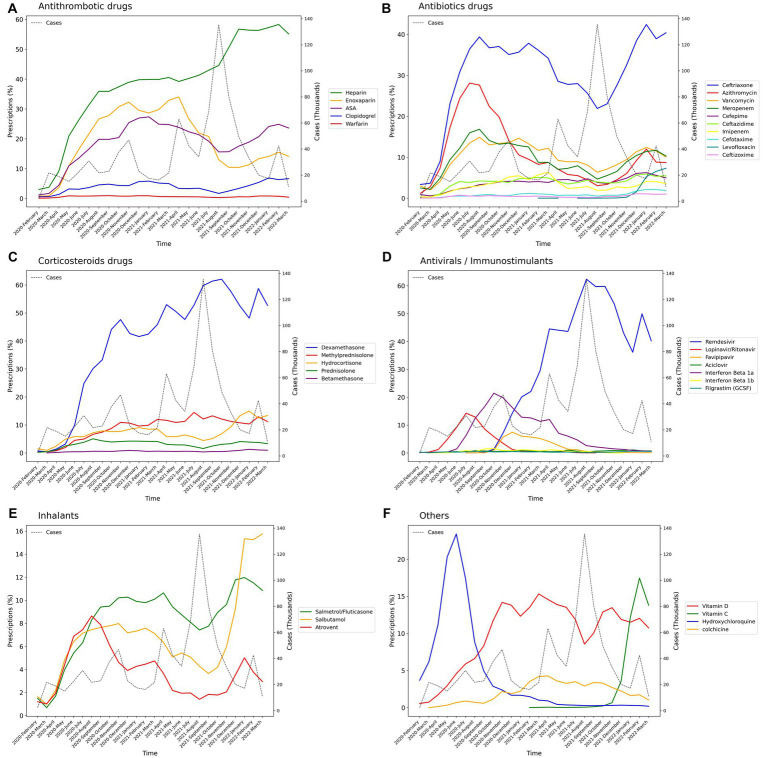
Percentage of medication prescriptions trends of drugs in each medication group during the study period (Lines in dots is the trend of the number of hospitalized COVID-19 cases). **(A)** Antithrombotics, **(B)** Antibiotics, **(C)** Corticosteroids, **(D)** Antivirals, **(E)** Inhalants, **(F)** Others.

Among the main groups of medications, antithrombotics (55.84% [95% CI: 55.74–55.94]), corticosteroids (54.14% [54.04–54.24]), and antibiotics (42.22% [42.12–42.32]) were the top used medications among cases with COVID-19. Investigating each group showed among antithrombotics, heparin (42.15% [42.05–42.25]); among corticosteroids, dexamethasone (47.45% [47.35–47.55]); and among antibiotics, ceftriaxone (28.95% [28.86–29.04]) was the most used drug. The most used antiviral medication was remdesivir (37.02% [36.92–37.12]). Among chronically used medications, atorvastatin (21.85% [21.76–21.93]), insulins (10.18% [10.11–10.24]), and losartan (9.01% [8.95–9.07]) were highly used by admitted patients (Table 1).However, there were some trivial significant differences in medication use between the two sexes, its pattern was almost similar between them in most medication groups (Table 2 and Supplementary Figure S1). Considering age groups, ACE inhibitors/ARB, antithrombotics, diuretics, and GI medications had an increasing pattern with the aging trend of the cases. Antibiotics were used with a higher frequency in two extremities of age (<10 (59.58% [59.08–60.07]) and > 80 age groups (53.94% [53.64–54.24])), while antivirals were more used in the 31–40 (48.05% [47.76–48.34]) and 41–50 (48.02% [47.75–48.29]) age groups (Table 2 and Supplementary Figure S1). Drug prescription patterns had a generally similar pattern among provinces but with some variations based on the groups of medications (Figure 1 and Supplementary Table S1). Each medical specialty had its drug prescription pattern based on the main drug groups (Table 2 and Supplementary Figure S2). For example, pediatricians prescribed antibiotics in 57.39% (95% CI: 56.78–58.00) of their cases, which was significantly more frequent compared to other specialties, and cardiologists had the highest prescription proportion for antithrombotics and ACE inhibitors/ARB, 66.10% (65.18–67.02) and 36.07% (35.14–37.00) of their cases, respectively, compared to any other specialties. The overall trend of medication use for almost all medication groups increased in the study period; however, the drug trends in each medication group showed diverse patterns (Figures 2, 3).

**Table 2:** Medication prescription percentage with 95% confidence interval among cases with COVID-19 for different main groups of drugs based on sex, age groups, and physician specialties.

	Antibiotics	ACE inhibitor/ARB	Antithrombotics	Antivirals	Corticosteroids	Vitamin D3	Antidiabetics	Diuretics	Gastrointestinal drugs	Hydroxychloroquine	Immunostimulants	Inhalants	Colchicine
Sex													
Female	41.06% (40.92–41.20)	14.31% (14.21–14.41)	56.17% (56.03–56.31)	41.29% (41.15–41.43)	54.50% (54.36–54.64)	10.78% (10.70–10.87)	12.97% (12.88–13.07)	11.39% (11.30–11.48)	41.54% (41.40–41.68)	2.79% (2.74–2.83)	7.25% (7.18–7.33)	10.16% (10.07–10.24)	2.53% (2.48–2.57)
Male	43.47% (43.32–43.62)	11.39% (11.30–11.48)	55.49% (55.34–55.64)	38.58% (38.44–38.73)	53.75% (53.60–53.89)	10.61% (10.52–10.70)	10.05% (9.97–10.14)	11.95% (11.86–12.05)	41.60% (41.46–41.75)	2.84% (2.80–2.89)	7.27% (7.19–7.35)	10.84% (10.75–10.93)	2.50% (2.46–2.55)
Age groups													
<10	59.58% (59.08–60.07)	1.15% (1.04–1.26)	22.00% (21.58–22.42)	4.97% (4.75–5.19)	25.85% (25.40–26.29)	2.05% (1.90–2.19)	0.71% (0.63–0.80)	5.31% (5.08–5.54)	20.29% (19.88–20.70)	0.75% (0.67–0.84)	1.11% (1.00–1.21)	3.74% (3.54–3.93)	0.07% (0.04–0.10)
11–20	38.36% (37.61–39.11)	1.46% (1.28–1.65)	32.86% (32.14–33.58)	24.00% (23.34–24.65)	35.65% (34.92–36.39)	6.23% (5.86–6.60)	2.29% (2.06–2.52)	4.44% (4.12–4.75)	29.90% (29.20–30.60)	1.81% (1.61–2.02)	3.27% (3.00–3.54)	4.06% (3.76–4.36)	0.64% (0.52–0.77)
21–30	32.40% (32.01–32.79)	1.55% (1.44–1.65)	46.36% (45.95–46.78)	41.14% (40.73–41.55)	48.48% (48.07–48.90)	8.64% (8.40–8.87)	3.54% (3.39–3.69)	3.14% (3.00–3.29)	33.64% (33.25–34.03)	2.40% (2.28–2.53)	4.92% (4.74–5.10)	6.13% (5.93–6.33)	1.59% (1.48–1.69)
31–40	34.06% (33.78–34.33)	2.59% (2.49–2.68)	52.42% (52.13–52.71)	48.05% (47.76–48.34)	55.85% (55.56–56.14)	10.37% (10.20–10.55)	5.92% (5.78–6.06)	4.16% (4.05–4.28)	37.15% (36.87–37.43)	2.44% (2.35–2.53)	6.28% (6.14–6.42)	8.17% (8.01–8.32)	2.40% (2.31–2.49)
41–50	36.13% (35.87–36.39)	5.97% (5.84–6.09)	55.20% (54.93–55.47)	48.02% (47.75–48.29)	58.17% (57.90–58.43)	11.18% (11.01–11.35)	9.55% (9.39–9.70)	5.98% (5.85–6.10)	39.36% (39.10–39.63)	2.56% (2.47–2.64)	7.31% (7.17–7.45)	9.69% (9.53–9.85)	2.83% (2.75–2.92)
51–60	39.19% (38.95–39.42)	12.33% (12.17–12.50)	57.25% (57.01–57.49)	44.87% (44.63–45.12)	57.39% (57.14–57.63)	11.24% (11.08–11.39)	14.03% (13.86–14.20)	9.66% (9.51–9.80)	41.50% (41.26–41.74)	2.79% (2.71–2.87)	7.94% (7.81–8.08)	11.16% (11.00–11.31)	2.97% (2.89–3.06)
61–70	43.39% (43.16–43.62)	18.89% (18.71–19.07)	60.26% (60.03–60.49)	40.97% (40.74–41.20)	56.22% (55.99–56.45)	11.75% (11.60–11.90)	16.79% (16.61–16.96)	14.63% (14.46–14.80)	44.29% (44.06–44.52)	3.15% (3.07–3.23)	8.29% (8.16–8.42)	12.03% (11.87–12.18)	2.99% (2.91–3.07)
71–80	48.15% (47.87–48.43)	23.65% (23.41–23.89)	62.25% (61.98–62.53)	35.76% (35.49–36.03)	54.96% (54.68–55.24)	11.79% (11.61–11.97)	16.12% (15.92–16.33)	19.42% (19.20–19.64)	47.62% (47.33–47.90)	3.37% (3.27–3.47)	8.55% (8.39–8.70)	13.03% (12.84–13.22)	2.75% (2.66–2.84)
>80	53.94% (53.64–54.24)	22.99% (22.74–23.24)	63.79% (63.50–64.08)	31.41% (31.13–31.69)	53.70% (53.41–54.00)	11.47% (11.28–11.66)	12.04% (11.85–12.24)	23.64% (23.39–23.90)	51.02% (50.72–51.31)	3.47% (3.36–3.58)	8.03% (7.87–8.20)	13.09% (12.89–13.29)	2.14% (2.06–2.23)
Physician Specialties													
Anaesthesiologist	44.60% (42.90–46.30)	19.00% (17.65–20.34)	52.86% (51.15–54.57)	35.18% (33.54–36.82)	49.92% (48.21–51.64)	14.87% (13.65–16.09)	12.88% (11.73–14.03)	25.42% (23.93–26.91)	46.31% (44.60–48.02)	2.26% (1.75–2.77)	7.04% (6.16–7.91)	13.34% (12.17–14.50)	4.13% (3.45–4.81)
Cardiology	38.38% (37.44–39.33)	36.07% (35.14–37.00)	66.10% (65.18–67.02)	15.05% (14.36–15.75)	33.26% (32.34–34.17)	6.29% (5.81–6.76)	15.29% (14.59–15.99)	36.01% (35.08–36.95)	42.97% (42.00–43.93)	1.53% (1.30–1.77)	5.65% (5.20–6.09)	8.11% (7.57–8.64)	2.22% (1.94–2.51)
Emergency	24.63% (24.20–25.06)	6.08% (5.84–6.32)	38.64% (38.16–39.13)	33.64% (33.16–34.11)	42.07% (41.57–42.56)	5.30% (5.07–5.52)	5.96% (5.72–6.19)	7.05% (6.79–7.30)	26.20% (25.76–26.64)	2.03% (1.89–2.17)	1.86% (1.72–1.99)	3.87% (3.68–4.06)	0.97% (0.87–1.07)
GP	42.32% (42.16–42.48)	12.48% (12.37–12.59)	55.71% (55.55–55.87)	39.92% (39.76–40.08)	54.99% (54.83–55.15)	10.19% (10.09–10.29)	10.67% (10.57–10.77)	11.08% (10.98–11.18)	41.40% (41.24–41.56)	2.36% (2.31–2.41)	7.05% (6.97–7.14)	10.36% (10.26–10.46)	2.31% (2.27–2.36)
Infectious	41.59% (41.35–41.84)	12.89% (12.72–13.05)	61.06% (60.81–61.30)	49.47% (49.22–49.72)	59.55% (59.30–59.79)	12.46% (12.30–12.62)	13.59% (13.42–13.76)	9.92% (9.78–10.07)	42.76% (42.52–43.01)	3.71% (3.62–3.81)	9.35% (9.20–9.49)	12.49% (12.32–12.65)	3.62% (3.53–3.71)
Internal	44.76% (44.51–45.00)	14.27% (14.09–14.44)	59.05% (58.81–59.30)	40.65% (40.40–40.89)	55.17% (54.92–55.42)	11.88% (11.72–12.04)	13.19% (13.02–13.36)	12.95% (12.79–13.12)	46.45% (46.20–46.70)	3.07% (2.98–3.15)	8.63% (8.49–8.77)	10.59% (10.44–10.75)	2.13% (2.06–2.21)
Pediatrics	57.39% (56.78–58.00)	3.14% (2.93–3.36)	24.68% (24.15–25.21)	6.78% (6.47–7.09)	26.27% (25.73–26.81)	2.61% (2.42–2.81)	1.71% (1.55–1.87)	7.58% (7.26–7.91)	23.53% (23.01–24.06)	0.93% (0.81–1.04)	1.47% (1.32–1.61)	3.97% (3.72–4.21)	0.21% (0.16–0.27)
Pulmonologist	45.59% (44.57–46.61)	15.30% (14.56–16.03)	58.94% (57.93–59.95)	35.09% (34.12–36.07)	56.03% (55.02–57.05)	16.28% (15.52–17.03)	14.44% (13.72–15.16)	16.29% (15.53–17.04)	40.41% (39.40–41.41)	2.45% (2.13–2.77)	6.05% (5.57–6.54)	10.93% (10.29–11.57)	2.62% (2.30–2.95)
Others	37.16% (36.49–37.83)	9.63% (9.22–10.04)	44.96% (44.27–45.65)	14.99% (14.49–15.49)	30.89% (30.25–31.53)	7.95% (7.58–8.33)	7.60% (7.23–7.97)	11.53% (11.08–11.97)	36.30% (35.63–36.97)	2.07% (1.88–2.27)	3.25% (3.01–3.50)	4.26% (3.98–4.54)	1.24% (1.08–1.39)
Unknown	42.68% (42.42–42.94)	14.68% (14.50–14.87)	57.91% (57.65–58.17)	42.47% (42.21–42.73)	57.97% (57.71–58.23)	11.98% (11.81–12.16)	13.28% (13.11–13.46)	13.32% (13.15–13.50)	43.42% (43.16–43.68)	3.51% (3.41–3.61)	7.27% (7.14–7.41)	12.43% (12.26–12.61)	3.24% (3.15–3.33)

Data in parenthesis are 95% confidence interval.

### 3.3 Medications contribution to hospitalization duration

**Figure 4:**
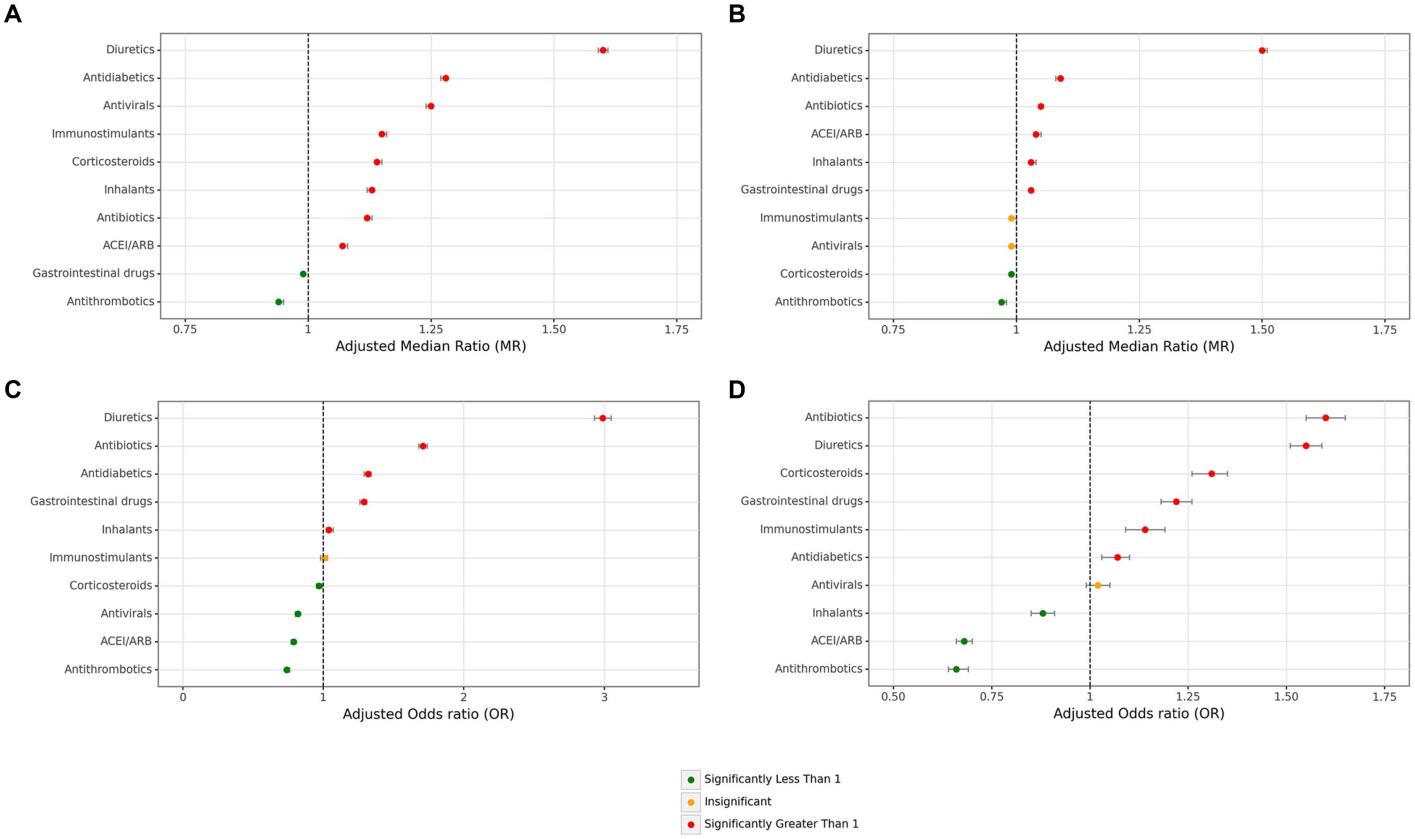
Associations between main medication groups and hospitalization stay and mortality. **(A)** Overall hospitalization duration days, **(B)** ICU duration days, **(C)** all cases mortality, **(D)** ICU-admitted cases mortality (ICU: intensive care unit. Adjustments were done with sex, age, month of admission, province, insurance fund type, admission type, physician specialty, other main groups of medications + Atorvastatin, Vitamin D, Hydroxychloroquine, Colchicine, and Nitroglycerin in this figure).

Investigation of the duration of hospitalization based on main medication groups showed antithrombotics (adjusted MR = 0.94 [95% CI: 0.94–0.95]) and marginally GI medications (0.99 [0.99–0.99]) were significantly associated with a shorter period of overall hospitalization. Other main groups of medications were associated with longer hospitalization periods, with the most remarkable effect sizes for diuretics (1.60 [1.59–1.61]), followed by anti-diabetics (1.28 [1.27–1.28]) and antivirals (1.25 [1.24–1.25]) (Figure 4A and Supplementary Table S2). Regarding the duration of hospitalization, except for immunostimulants and anti virals which had no significant impact on ICU hospitalization period, diuretics (1.50 [1.50–1.51]), anti-diabetics (1.09 [1.08–1.09]), antibiotics, ACE inhibitor/ARB, inhalants, and GI drug were associated with significantly longer stay at ICU with only remarkable effect size in the first group, in contrast to antithrombotics (0.97 [0.97–0.98]) and corticosteroids (0.99 [0.99–0.99]) (Figure 4B and Supplementary Table S2).

**Figure 5:**
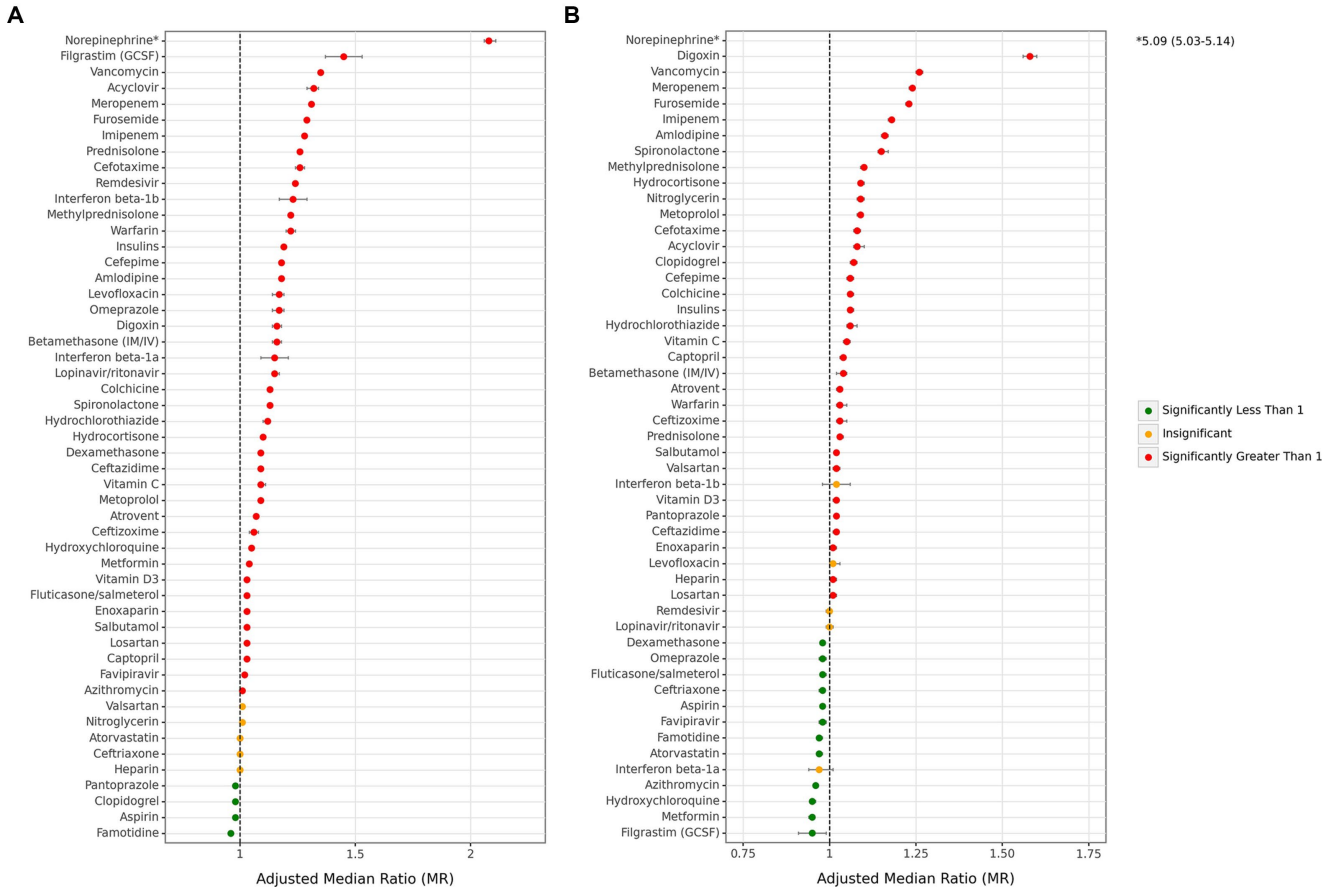
Associations between each individual medication and median hospitalization stay. **(A)** Overall hospitalization duration days, **(B)** ICU duration days. (ICU: intensive care unit. Adjustments were done with sex, age, month of admission, province, insurance fund type, admission type, physician specialty, and other drugs in this figure).

There were variations in the associations of medications from each main drug group with overall hospitalization and ICU stay durations. The majority of drugs were associated with an increase in these durations, while a limited number of drugs with a small effect size were associated with a decrease. Notably, famotidine and filgrastim (GCSF) exhibited the most protective associations with total hospitalization and ICU days, resulting in decreased durations of 0.96 (0.95–0.96) and 0.95 (0.91–0.99), respectively (Figure 5 and Supplementary Table S3). In contrast, norepinephrine showed the highest association with both overall hospitalization (2.08 [2.06–2.11]) and ICU stay durations (5.09 [5.03–5.14]) compared to other drugs. Among antithrombotics, warfarin had the most significant effect on the total hospitalization period in all cases (1.22 [1.20–1.24]). However, other agents did not show a notable association with it. When considering ICU stay duration, only clopidogrel (1.07 [1.06–1.08]) displayed a remarkably significant association. Among antibiotics, vancomycin (total: 1.35 [1.34–1.36], ICU: 1.26 [1.25–1.27]), meropenem (total: 1.31 [1.31–1.32], ICU: 1.24 [1.23–1.24]), and imipenem (total: 1.28 [1.27–1.29], ICU: 1.18 [1.17–1.18]) were the top three antibiotics associated with increased duration of total hospital and ICU stays. In contrast, no antibiotics were associated with a decrease in total hospitalization duration. However, azithromycin (0.96 [0.96–0.96]) and ceftriaxone (0.98 [0.97–0.98]) showed a small association with decreased ICU duration. Among corticosteroids, only dexamethasone was marginally associated with a decrease in ICU duration (0.98 [0.98–0.98]), while other corticosteroids were associated with an increase in hospital and ICU stays. While all antivirals were not associated with ICU duration days, acyclovir and remdesivir were remarkably associated with an increase in total hospitalization days by 1.32 (1.29–1.34) and 1.24 (1.24–1.25), respectively.

### 3.4 Medications contribution to mortality

Evaluation of associations with mortality rates among all cases according to medication groups, antithrombotics (adjusted OR = 0.74 [95% CI: 0.73–0.76]), ACE inhibitor/ARB (0.79 [0.77–0.80]), antivirals (0.82 [0.80–0.83]), and corticosteroids (0.97 [0.95–0.99]), were significantly associated with lower mortality. In contrast, diuretics with the most significant effect size (2.99 [2.93–3.05]), followed by antibiotics (1.71 [1.68–1.74]), antidiabetics (1.32 [1.29–1.34]), and GI medications (1.29 [1.26–1.31]) (Figure 4C and Supplementary Table S4). Investigation on mortality rates in cases admitted to ICU revealed only three groups of antithrombotics (0.66 [0.64–0.69]), ACE inhibitor/ARB (0.68 [0.66–0.70]), inhalants (0.88 [0.85–0.91]) were protective associated with mortality, while antibiotics (1.60 [1.55–1.65]), diuretics (1.55 [1.51–1.59]), and corticosteroids (1.31 [1.26–1.35]) were the medication groups with the greatest risk for mortality (Figure 4D and Supplementary Table S4).

**Figure 6:**
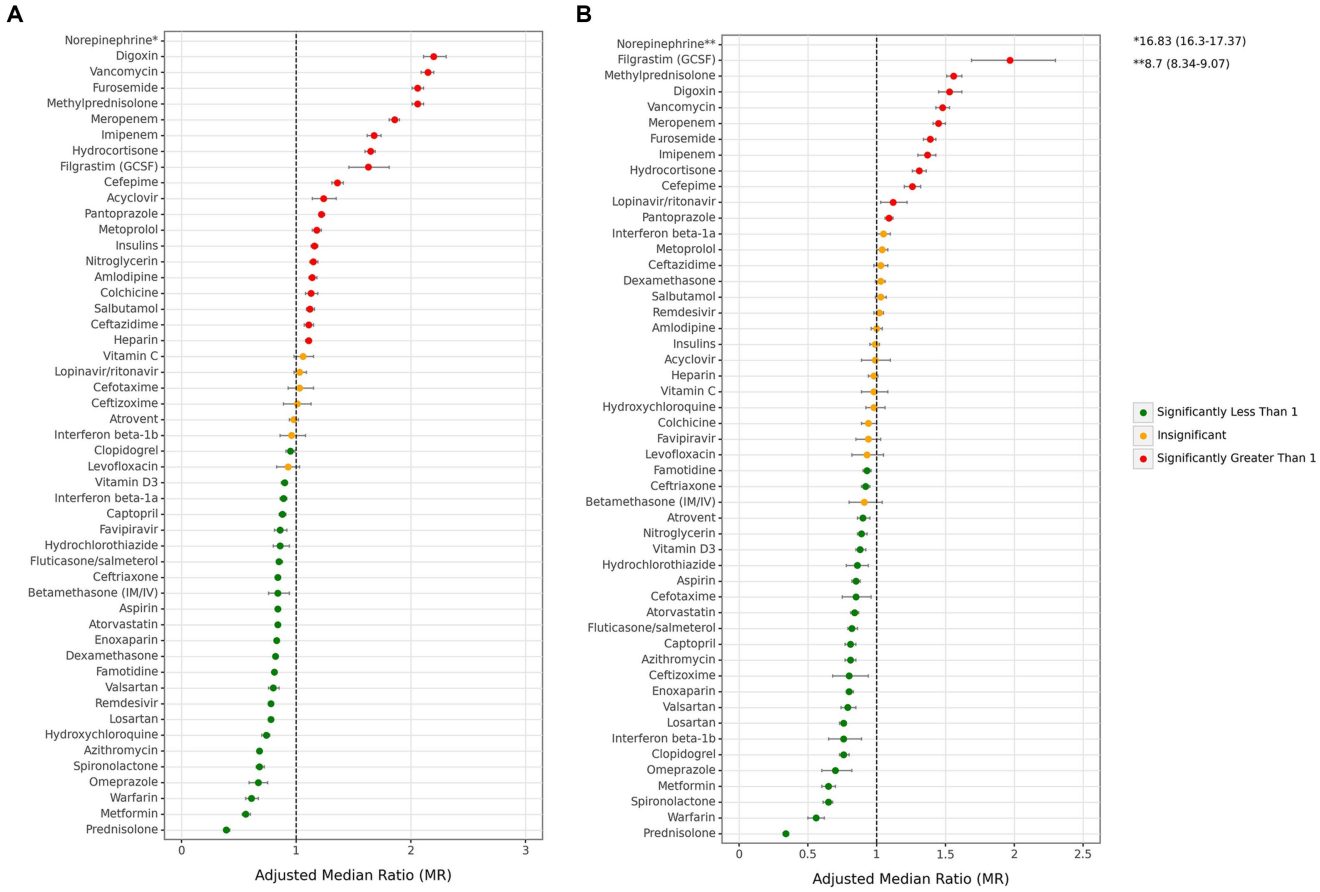
Associations between each individual medication and mortality. **(A)** All cases, **(B)** ICU-admitted cases. (ICU: intensive care unit. Adjustments were done with sex, age, month of admission, province, insurance fund type, admission type, physician specialty, and other drugs in this figure).

Considering medications within each main drug group, there are variations in the association of medications in some groups with mortality (Figure 6 and Supplementary Table S5). Among antithrombotics, warfarin was associated with the most protection against mortality both in all cases (0.61 [0.56–0.67]) and ICU admitted cases (0.56 [0.50–0.62]). In contrast, heparin was the only drug in this group related to increased mortality in all cases (1.11 [1.08–1.13]) and was not associated with mortality in ICU-admitted cases (0.98 [0.94–1.01]).Among antibiotics, vancomycin was a remarkably greater risk factor for mortality both in all cases (2.15 [2.09–2.20]), and ICU admitted cases (1.48 [1.43–4.53]), while azithromycin and ceftriaxone were the only antibiotics which were associated to lower mortality for both of the all (azithromycin: 0.68 [0.66–0.7], ceftriaxone 0.84 [0.82–0.86]) and ICU cases (azithromycin: 0.81 [0.77–0.85], ceftriaxone 0.92 [0.89–0.95]). Among corticosteroids, prednisolone was associated with the highest protection against mortality in all cases (0.39 [0.37–0.42]) and ICU cases (0.34 [0.32–0.36]). It also offered the most protection among all drugs. In contrast to prednisolone, methylprednisolone was one of the drugs with highest association with mortality in both all (2.06 [2.01–2.11]) and ICU cases (1.56 [1.51–1.62]).Among antivirals, remdesivir was associated with the most reduction in mortality in all cases (0.78 [0.76–0.80]), while no antiviral drug was associated with mortality in ICU cases. All ACE inhibitors/ARB drugs were associated with a decrease in mortality in both all and ICU cases. When considering antidiabetic drugs, metformin had one of the highest associations with the reduction of mortality in all (0.56 [0.53–0.60]) and ICU cases (0.65 [0.60–0.70]). In contrast, insulin was associated with an increase in mortality in all cases (1.16 [1.13–1.19]) and showed no association in ICU cases. Considering remaining drugs, norepinephrine (all: 16.83 [16.3–17.37], ICU: 8.7 [8.34–9.07]) was remarkably associated with a higher risk of mortality in comparison to filgrastim (GCSF), digoxin, and furosemide, which were the other drugs with the highest associations with mortality in both all and ICU-admitted cases.

## 4 Discussion

This study investigated the drug prescription patterns among COVID-19 patients with IHIO insurance during a 26-month period and assessed the contribution of different groups of medications to hospital stay and mortality. The main findings of this study were antithrombotics, corticosteroids, and antibiotics as the top groups of medications used for patients, similar prescription patterns for both sexes, specific patterns of drug prescription by clinical specialties, the remarkable association of antithrombotics use and shorter hospital and ICU stay, significant protective association of antithrombotics, corticosteroids, and antivirals against mortality due to COVID-19, and higher risk of mortality with prescription of diuretics, antibiotics, and antidiabetics. It was also concluded that while a minority of drugs had a minor association with reducing hospitalization stays, a wider range of drugs were significantly associated with a decrease in COVID-19 mortality. This highlights that the primary association of medication in this study was with reducing mortality rather than reducing the length of hospital stay, which were the two primary outcomes under investigation.

Besides reporting the most used medications for COVID-19, this study successfully pictured the trends of medication use over the long period of assessment, comparable with similar literature and national guidelines on the use of medications for these patients. This study found decreasing trends in the use of antibiotics and hydroxychloroquine and increasing trends in the use of corticosteroids and remdesivir by progress in the study period which is in alignment with changes in national COVID-19 treatment guidelines in Iran (24). Similar findings have been reported in studies on hospitalized patients with COVID-19 in the United States, highlighting the increasing interest and utilization of dexamethasone and remdesivir. However, there were variations between institutions and populations like those of different races (25, 26). Although the use of antibiotics declined during the study period, some types of this category, like ceftriaxone, had a growing use which could be due to bacterial co-infection in patients with COVID-19; however, the declining use of azithromycin as a widely prescribed antibiotic in Iran was indicative of abolishment of unnecessary use of antibiotics for this infection as the body of evidence showed this treatment approach was not effective in the case of COVID-19 (27, 28).

One of the medication groups with the highest benefits in this study was antithrombotics, which were associated with a 6% reduction in total hospital stays and a 26% decrease in mortality rates for all cases hospitalized with COVID-19, respectively. Although the general findings on antithrombotics showed advantages, some medications in this group, like heparin, showed inconsistent relationships. Heterogeneity about the effects of antithrombotics on COVID-19 could be found in similar publications. No significant reduction in mortality rates were found in a meta-analysis on the use of therapeutic and prophylactic doses of antithrombotics in COVID-19 patients (29). In contrast to our findings, in a cohort study from Italy, heparin could reduce the risk of COVID-19-associated mortality by up to 40%, and this impact was more substantial in cases with severe infection (30). In a systematic review of antithrombotics’ effects on COVID-19 mortality, three studies reported lower mortality, while six studies could not show any significant effects (31). As a higher level of evidence, a clinical trial also reported no statistically significant protective effects of antithrombotics in COVID-19 patients (32). On the other hand, pooling evidence in two meta-analyses showed a reduction in thrombotic events (33) and mortality in hospitalized patients (34). The inconsistencies between studies on different effects of antithrombotics on COVID-19 could be justified by differences in types of used antithrombotics, dose variations, epidemiologic and genetic differences between populations of patients, and changes in COVID-19 virus genetics through time (35).

Overall, the antibiotic prescription in this study was associated with a higher probability of mortality by 71 and 60% in all cases and ICU-admitted cases, and longer hospitalization periods by 12 and 5% total hospitalization and ICU duration. Although this finding is derived from the real-world data of hospitalized COVID-19 patients, interpreting the results should be done cautiously since, generally, patients with severe conditions are prone to co-infections and are highly at risk of adverse disease outcomes like death. This statement is more rational when inspecting the types of antibiotics individually. As reported in the previous section, strong antibiotics like vancomycin were noticeably associated with longer hospitalization and higher mortalities. Evaluating the literature also found similar findings on higher morbidity and mortality rates with antibiotics in COVID-19 (36, 37). It is essential to consider the effect of bacterial co-infection on COVID-19 patients’ survival; however, separating the effects of infection from antibiotics is somehow impossible. Therefore, the use of antibiotics in COVID-19 should be evidence-based, and appropriate guidelines should be referred during patient management (38). Another major concern about the use of antibiotics in the COVID-19 era is antimicrobial resistance, which might predispose the populations to a double burden of several resistant infections, and it must be considered before prescribing any antibiotic (39, 40).

One of the prominent findings of this study was the high frequency of use of corticosteroids in about 54% of cases and its association with disease outcomes, especially for dexamethasone and prednisolone, two drugs mentioned as the two main corticosteroids in the latest version of guidelines (24). These were associated with an 18 and 61% reduction in overall mortality, respectively. This finding was generally consistent with the body of evidence as a clinical trial reported similar protective effects of corticosteroids (OR = 0.83) on COVID-19 (41), and pooled meta-analysis showed the same effect size (OR = 0.88) (42). Furthermore, our study revealed that methylprednisolone and hydrocortisone, two other corticosteroids prescribed steadily in approximately 10% of cases during the study period, were associated with a higher risk of mortality, being 2.06 and 1.65 times higher, respectively. This could be attributed to their prescription in severe cases where patients did not respond to other drugs, as well as potential non-adherence of physicians to standard guidelines. However, in patients with previous chronic use of corticosteroids, this medication group for COVID-19 resulted in higher mortality rates, especially in chronic use of dexamethasone (14). As an agent alleviating the vigorous inflammatory state and cytokine storm during the COVID-19 infection, corticosteroids are more effective in patients with severe infection and those who need mechanical ventilation support (43). Despite these findings, using corticosteroids for COVID-19 has specific pros and cons that should be considered in the long term to achieve the best treatment results (44).

Among antiviral agents, remdesivir was among the first to receive international approvals for COVID-19, which our study showed to have a protective association with COVID-19 case mortality by 22%, although it was associated with longer hospital stay by 24%. On the other hand, the protective association of this agent could not be inferred from sub-group analysis on patients admitted to ICU. One justification for this finding could be the maximum effectivity of this agent on mild to moderate cases of disease and less noticeable effects on severe cases, as it has been recommended as the indication for prescribing remdesivir in the national guideline (24). The first clinical trial of using remdesivir for COVID-19 reported an effect size of OR = 0.73 (45). This finding was consistent with a meta-analysis of studies that reported this agent could decrease the mortality rates of COVID-19 by 30% (46). On the other hand, another pooled meta-analysis of studies reported that remdesivir was not significantly effective on patients with a history of mechanic ventilation support and had mild protective effects against COVID-19 mortality (47). A recent meta-analysis published in 2023 showed that remdesivir had little or no effect on all-cause mortality or in-hospital mortality of individuals with moderate to severe COVID-19 (48). Therefore, future use of this agent needs further research and validation.

This study revealed that the use of certain medications related to chronic diseases is associated with a reduction in the risk of mortality. Previous studies have shown that the risk of mortality in COVID-19 patients with kidney diseases, cerebrovascular diseases, cardiovascular diseases, respiratory diseases, diabetes, hypertension, and cancers is 4.9, 4.78, 4, 2.74, 1.97, 1.97, and 1.89 times higher, respectively (49). Considering diabetes and hypertension, two prevalent non-communicable diseases (NCDs) in Iran (3), antidiabetic medications and ACE inhibitors/ARBs, commonly used to treat hypertension, were associated with a 21% decrease and a 32% increase in the risk of mortality in all cases. In the antidiabetic category, it’s noteworthy that insulin usage played a primary role in increasing the risk of mortality, whereas metformin usage was linked to a 44% reduction in mortality in all cases. These findings suggest that mortality in diabetic patients is linked to the progression of their diabetes. Similar results have been observed in other studies. In the Malekpour et al. study, the use of metformin was associated with a 13% reduction in mortality (14), and in another meta-analysis, a 34% reduction in mortality was reported (50). Concerning hypertension treatment, drugs like losartan, captopril, and valsartan from the ACE inhibitor/ARB class were associated with a reduction in mortality, consistent with previous studies (14, 51). Also, it is well investigated that hypertension is strongly associated with severe COVID-19 and could significantly increase the chance of adverse disease outcomes like death (5, 52, 53). Some anti-hypertensive medications like ACE inhibitors/ARBs impact COVID-19 further than controlling high blood pressure, which goes back to the infection mechanisms relying on the renin–angiotensin system (RAS) (54, 55). Among the other most chronically used medications for NCDs, atorvastatin and aspirin were also associated with lower mortality rates due to COVID-19 in this study, both by 16%. As investigated in the literature, continuing the treatment of underlying comorbidities like NCDs during a COVID-19 infection might help control the infection and positively impact the disease management course (14). Therefore, considering the bilateral connection between COVID-19 and chronic diseases like NCDs (5), considering the necessary medications to control these comorbidities while handling COVID-19 patients is highly suggested.

This study had some limitations. The prominent limitation of the study was the data source used to investigate the aims as a registry may have several challenges in recording data, including various degrees of missing values. Other limitations regarding the IHIO database might be a lack of data on medications that are not under insurance coverage, lack of access to other medications routinely used by hospitalized patients which might not be prescribed and recorded during their admission, and exclusion of different insurance types in this study that might jeopardize generalization of the findings of this study. Furthermore, considering that the data is related to Iran, it would limit the results of this study to developing countries and health systems with a quality of care profile similar to that of Iran. In addition, the clinical outcome of COVID-19 is associated with the severity of the disease and its clinical symptoms, which are not provided in our dataset. However, we attempted to account for this by adjusting for variables such as month, admission type, physician specialty, and medications, which are related to disease severity and underlying conditions in our analyses. Besides all these limitations, this study had several strengths, including having real-world data on medications prescribed for COVID-19 patients admitted at hospitals with a robust quality since the recorded data is free of self-reported biases like recall bias. Also, the robust inclusion of patients and cleaning the data by defining cases based on strict criteria were the other strengths of this study. Also, this study is among the first attempts to analyze and report claims data in Iran. The study design and methods could be used for similar future research in the country with promising results.

## 5 Conclusion

Based on the claims data recorded and provided by IHIO, this study reported antithrombotics, corticosteroids, and antibiotics as the top groups of medications used for patients hospitalized with COVID-19 during more than 2 years of investigation. Trends in medication prescription varied based on various factors across the country. Also, the investigated associations between various medications and duration of hospital stay and mortality due to COVID-19 pave the way for the development of current guidelines on the management of this infection. Benefitting from this experience, medication prescriptions could significantly impact mortality and hospitalization trends during epidemics and pandemics, affecting health and economic burdens. Therefore, they require special consideration during public health emergencies of infectious types.

## Data availability statement

The datasets presented in this article are not readily available because they are obtained from the IHIO registry, and due to privacy concerns for the patients in this registry, authors are not permitted to share the data publicly or privately. However, any researcher with written permission can request to obtain the anonymized data. Requests to access the datasets should be directed to IHIO website.

## Ethics statement

This study was approved by the ethical committee at the School of Public Health, Tehran University of Medical Sciences (code: IR.TUMS.SPH.REC.1401.120). The provided data by IHIO in this study were de-identified and data holder and study investigators were responsible to save the privacy of the patients and users of the IHIO insurance services. The studies were conducted in accordance with the local legislation and institutional requirements. Written informed consent for participation was not required from the participants or the participants’ legal guardians/next of kin in accordance with the national legislation and institutional requirements.

## Author contributions

RM: Conceptualization, Methodology, Writing – original draft, Writing – review & editing. AG: Conceptualization, Data curation, Formal analysis, Methodology, Visualization, Writing – original draft, Writing – review & editing. M-RM: Data curation, Methodology, Writing – review & editing. HK: Writing – original draft, Writing – review & editing. MN: Validation, Writing – review & editing. ME: Validation, Writing – review & editing. HR: Validation, Writing – review & editing. ZS: Validation, Writing – review & editing. AS: Methodology, Supervision, Validation, Writing – review & editing. RD: Conceptualization, Methodology, Project administration, Supervision, Validation, Writing – review & editing.

## Footnotes

^1^https://www.python.org/

^2^https://cran.r-project.org/
