# Supplementary material for: Drug prescription patterns and their association with mortality and hospitalization duration in COVID-19 patients: insights from big data
Source: Front Public Health. 2023 Dec 18;11:1280434. doi: 10.3389/fpubh.2023.1280434 (PMC10758044; doi:10.3389/fpubh.2023.1280434)
Supplement: Supplementary file 1 [file Data_Sheet_1.docx]

**Supplementary Table 1.** Percentage of medication prescriptions with 95% confidence interval among cases with COVID-19 for different main groups of drugs in provinces of Iran.

| Province | ACE inhibitor/ARB | Antibiotics | Antidiabetics | Antithrombotics | Antivirals | Colchicine | Corticosteroids | Diuretics | Gastrointestinal Drugs | Hydroxychloroquine | Immunostimulants | Inhalants | Vitamin D3 |
| --- | --- | --- | --- | --- | --- | --- | --- | --- | --- | --- | --- | --- | --- |
| Alborz | 5.59% (5.25-5.93) | 11.11% (10.64-11.57) | 3.93% (3.64-4.22) | 19.64% (19.05-20.23) | 26.50% (25.84-27.15) | 1.54% (1.35-1.72) | 27.60% (26.94-28.26) | 3.97% (3.68-4.26) | 21.19% (20.59-21.80) | 0.28% (0.20-0.36) | 1.24% (1.07-1.40) | 1.63% (1.44-1.81) | 2.90% (2.65-3.15) |
| Ardabil | 14.63% (14.12-15.13) | 48.01% (47.29-48.73) | 13.36% (12.87-13.85) | 62.38% (61.69-63.08) | 32.53% (31.86-33.21) | 0.81% (0.68-0.94) | 49.03% (48.31-49.75) | 8.69% (8.28-9.09) | 41.78% (41.08-42.49) | 2.24% (2.03-2.45) | 5.98% (5.64-6.32) | 19.38% (18.81-19.94) | 11.29% (10.83-11.74) |
| Azerbaijan, East | 10.31% (10.06-10.56) | 23.46% (23.11-23.81) | 6.01% (5.82-6.21) | 33.23% (32.84-33.61) | 19.49% (19.16-19.81) | 1.04% (0.96-1.12) | 34.56% (34.17-34.95) | 7.16% (6.94-7.37) | 24.39% (24.04-24.74) | 0.74% (0.67-0.81) | 5.58% (5.40-5.77) | 8.49% (8.26-8.72) | 7.23% (7.02-7.44) |
| Azerbaijan, West | 11.56% (11.28-11.84) | 33.72% (33.31-34.14) | 13.54% (13.24-13.84) | 54.10% (53.67-54.54) | 32.04% (31.63-32.45) | 1.70% (1.59-1.82) | 54.73% (54.29-55.16) | 8.68% (8.43-8.93) | 48.66% (48.22-49.10) | 3.02% (2.87-3.17) | 5.64% (5.44-5.85) | 10.49% (10.22-10.76) | 10.84% (10.57-11.11) |
| Bushehr | 11.55% (10.86-12.23) | 57.39% (56.33-58.44) | 25.87% (24.93-26.80) | 72.10% (71.14-73.06) | 46.16% (45.10-47.23) | 3.67% (3.27-4.07) | 70.43% (69.45-71.40) | 10.82% (10.16-11.49) | 60.75% (59.70-61.79) | 5.49% (5.01-5.98) | 12.71% (12.00-13.42) | 12.14% (11.44-12.84) | 27.35% (26.40-28.30) |
| Chaharmahal and Bakhtiari | 21.40% (20.70-22.10) | 56.53% (55.68-57.37) | 13.45% (12.86-14.03) | 78.73% (78.03-79.43) | 39.28% (38.45-40.11) | 4.30% (3.95-4.65) | 74.75% (74.01-75.49) | 16.07% (15.44-16.69) | 53.04% (52.18-53.89) | 2.65% (2.38-2.92) | 8.59% (8.12-9.07) | 22.15% (21.44-22.85) | 17.86% (17.21-18.51) |
| Fars | 11.98% (11.71-12.25) | 41.78% (41.38-42.19) | 7.01% (6.80-7.22) | 61.44% (61.04-61.84) | 58.98% (58.58-59.39) | 2.57% (2.44-2.70) | 54.18% (53.77-54.59) | 10.12% (9.87-10.37) | 40.56% (40.16-40.97) | 2.98% (2.84-3.12) | 4.28% (4.12-4.45) | 15.82% (15.52-16.12) | 16.14% (15.84-16.44) |
| Gilan | 5.93% (5.64-6.22) | 34.27% (33.69-34.85) | 16.27% (15.82-16.72) | 48.72% (48.10-49.33) | 60.45% (59.85-61.05) | 0.49% (0.41-0.58) | 45.73% (45.12-46.34) | 8.67% (8.32-9.01) | 29.62% (29.06-30.18) | 1.74% (1.58-1.90) | 8.73% (8.38-9.07) | 3.68% (3.45-3.91) | 2.22% (2.04-2.40) |
| Golestan | 16.65% (16.20-17.10) | 67.29% (66.72-67.86) | 20.63% (20.14-21.12) | 58.39% (57.79-58.98) | 50.33% (49.72-50.93) | 2.26% (2.08-2.44) | 65.24% (64.66-65.81) | 20.04% (19.56-20.53) | 51.31% (50.70-51.91) | 4.18% (3.94-4.42) | 8.54% (8.20-8.88) | 14.18% (13.75-14.60) | 10.02% (9.66-10.39) |
| Hamadan | 16.38% (15.99-16.77) | 47.70% (47.17-48.22) | 15.88% (15.49-16.26) | 71.00% (70.52-71.48) | 68.07% (67.58-68.56) | 2.04% (1.89-2.19) | 76.13% (75.68-76.58) | 11.02% (10.69-11.35) | 49.28% (48.76-49.81) | 3.78% (3.58-3.98) | 25.85% (25.38-26.31) | 30.28% (29.79-30.76) | 7.44% (7.16-7.72) |
| Hormozgan | 6.96% (6.63-7.29) | 37.06% (36.44-37.68) | 14.65% (14.20-15.11) | 59.77% (59.14-60.40) | 43.14% (42.51-43.78) | 2.56% (2.36-2.76) | 53.33% (52.70-53.97) | 4.58% (4.31-4.84) | 41.22% (40.59-41.85) | 1.49% (1.33-1.65) | 11.68% (11.27-12.09) | 4.88% (4.60-5.16) | 5.08% (4.80-5.37) |
| Ilam | 12.66% (12.09-13.23) | 47.72% (46.86-48.58) | 5.31% (4.92-5.69) | 58.59% (57.74-59.43) | 63.48% (62.65-64.31) | 3.12% (2.82-3.42) | 70.87% (70.09-71.65) | 7.15% (6.70-7.59) | 41.41% (40.56-42.25) | 3.79% (3.46-4.11) | 6.91% (6.47-7.34) | 6.32% (5.90-6.74) | 25.31% (24.57-26.06) |
| Isfahan | 21.97% (21.62-22.32) | 45.66% (45.24-46.08) | 17.42% (17.10-17.74) | 74.08% (73.71-74.45) | 33.71% (33.31-34.10) | 2.56% (2.43-2.69) | 67.91% (67.51-68.30) | 28.14% (27.76-28.52) | 61.84% (61.44-62.25) | 3.69% (3.53-3.85) | 4.83% (4.65-5.01) | 10.97% (10.70-11.23) | 11.16% (10.90-11.43) |
| Kerman | 9.64% (9.36-9.91) | 40.36% (39.91-40.81) | 4.19% (4.01-4.38) | 49.09% (48.63-49.55) | 31.77% (31.34-32.20) | 1.47% (1.36-1.58) | 47.02% (46.56-47.48) | 9.64% (9.37-9.91) | 35.72% (35.28-36.16) | 1.30% (1.20-1.41) | 3.55% (3.38-3.72) | 8.94% (8.68-9.21) | 13.14% (12.83-13.45) |
| Kermanshah | 12.40% (12.01-12.79) | 49.89% (49.30-50.48) | 15.17% (14.75-15.59) | 59.20% (58.62-59.78) | 47.63% (47.04-48.21) | 13.95% (13.54-14.35) | 62.18% (61.61-62.75) | 7.39% (7.09-7.70) | 39.81% (39.24-40.39) | 4.08% (3.85-4.32) | 5.69% (5.41-5.96) | 3.29% (3.08-3.49) | 3.61% (3.39-3.83) |
| Khorasan, North | 17.86% (17.31-18.41) | 52.46% (51.73-53.18) | 11.78% (11.31-12.24) | 66.89% (66.22-67.57) | 58.24% (57.53-58.95) | 1.79% (1.60-1.98) | 68.47% (67.80-69.14) | 12.29% (11.81-12.76) | 37.86% (37.16-38.56) | 7.18% (6.81-7.55) | 15.62% (15.09-16.14) | 11.40% (10.94-11.86) | 5.20% (4.88-5.52) |
| Khorasan, Razavi | 13.63% (13.38-13.88) | 54.52% (54.16-54.89) | 15.04% (14.78-15.30) | 56.36% (55.99-56.72) | 30.29% (29.95-30.62) | 3.49% (3.36-3.62) | 54.92% (54.56-55.29) | 13.84% (13.58-14.09) | 44.11% (43.75-44.47) | 3.26% (3.13-3.39) | 4.01% (3.87-4.15) | 7.14% (6.95-7.33) | 6.94% (6.76-7.13) |
| Khorasan, South | 14.69% (14.11-15.26) | 58.21% (57.41-59.01) | 6.01% (5.63-6.40) | 47.64% (46.83-48.45) | 18.96% (18.32-19.60) | 1.49% (1.29-1.68) | 39.00% (38.21-39.79) | 10.09% (9.60-10.58) | 41.94% (41.14-42.74) | 0.94% (0.78-1.09) | 12.25% (11.71-12.78) | 15.01% (14.43-15.59) | 5.88% (5.49-6.26) |
| Khuzestan | 10.94% (10.67-11.21) | 40.51% (40.09-40.94) | 15.73% (15.41-16.04) | 62.03% (61.61-62.45) | 60.73% (60.31-61.15) | 1.00% (0.91-1.09) | 64.94% (64.53-65.35) | 9.89% (9.63-10.15) | 52.49% (52.06-52.92) | 3.04% (2.89-3.18) | 3.96% (3.79-4.12) | 10.87% (10.60-11.14) | 15.76% (15.44-16.07) |
| Kohgiluyeh and Boyer-Ahmad | 5.91% (5.50-6.33) | 34.07% (33.24-34.90) | 7.23% (6.77-7.68) | 47.13% (46.25-48.00) | 51.54% (50.67-52.42) | 1.03% (0.86-1.21) | 54.11% (53.24-54.99) | 3.84% (3.51-4.18) | 18.12% (17.45-18.80) | 0.44% (0.32-0.55) | 8.29% (7.80-8.77) | 21.23% (20.51-21.94) | 3.78% (3.45-4.11) |
| Kurdistan | 12.79% (12.35-13.22) | 28.70% (28.11-29.29) | 7.27% (6.93-7.61) | 68.31% (67.70-68.92) | 52.73% (52.08-53.39) | 5.16% (4.87-5.45) | 62.58% (61.95-63.21) | 7.94% (7.59-8.30) | 51.09% (50.43-51.74) | 1.66% (1.49-1.83) | 9.15% (8.78-9.53) | 17.91% (17.41-18.41) | 18.03% (17.53-18.53) |
| Lorestan | 10.89% (10.57-11.21) | 39.84% (39.34-40.34) | 8.74% (8.45-9.03) | 53.94% (53.43-54.45) | 36.66% (36.16-37.15) | 1.71% (1.58-1.85) | 51.43% (50.92-51.94) | 6.59% (6.34-6.85) | 33.22% (32.74-33.70) | 1.88% (1.74-2.02) | 6.28% (6.04-6.53) | 9.36% (9.06-9.65) | 11.31% (10.98-11.63) |
| Markazi | 10.51% (9.99-11.02) | 26.96% (26.22-27.71) | 6.91% (6.48-7.33) | 40.79% (39.96-41.61) | 27.76% (27.01-28.52) | 3.58% (3.27-3.89) | 39.17% (38.35-39.99) | 7.96% (7.50-8.41) | 27.60% (26.85-28.35) | 0.58% (0.45-0.71) | 4.15% (3.82-4.49) | 7.98% (7.52-8.43) | 8.92% (8.45-9.40) |
| Mazandaran | 15.12% (14.81-15.44) | 56.82% (56.39-57.26) | 11.27% (10.99-11.55) | 66.14% (65.72-66.56) | 46.65% (46.21-47.09) | 1.24% (1.14-1.33) | 60.89% (60.46-61.32) | 19.19% (18.84-19.53) | 50.89% (50.45-51.33) | 5.24% (5.04-5.44) | 11.51% (11.23-11.79) | 7.66% (7.43-7.90) | 7.98% (7.75-8.22) |
| Qazvin | 24.31% (23.53-25.09) | 54.10% (53.20-55.01) | 34.05% (33.19-34.91) | 76.71% (75.94-77.48) | 41.85% (40.96-42.75) | 4.18% (3.82-4.55) | 51.45% (50.54-52.36) | 19.72% (19.00-20.45) | 54.44% (53.53-55.34) | 6.86% (6.40-7.31) | 2.29% (2.02-2.57) | 19.59% (18.87-20.31) | 20.26% (19.53-20.99) |
| Qom | 6.93% (6.54-7.31) | 42.51% (41.76-43.26) | 14.48% (13.95-15.01) | 38.54% (37.80-39.27) | 37.36% (36.63-38.09) | 1.00% (0.85-1.15) | 36.02% (35.30-36.75) | 14.17% (13.65-14.70) | 32.96% (32.25-33.67) | 0.58% (0.47-0.70) | 14.61% (14.07-15.14) | 7.32% (6.93-7.71) | 7.67% (7.27-8.07) |
| Semnan | 21.05% (20.21-21.90) | 41.59% (40.57-42.60) | 18.10% (17.31-18.90) | 66.96% (65.99-67.93) | 35.25% (34.26-36.24) | 3.11% (2.75-3.47) | 65.99% (65.02-66.97) | 19.29% (18.47-20.10) | 37.74% (36.73-38.74) | 1.83% (1.55-2.11) | 5.45% (4.98-5.92) | 10.12% (9.50-10.74) | 25.36% (24.46-26.26) |
| Sistan and Baluchestan | 5.06% (4.72-5.40) | 29.08% (28.37-29.79) | 1.98% (1.76-2.19) | 32.55% (31.82-33.28) | 16.86% (16.27-17.44) | 0.13% (0.07-0.18) | 37.80% (37.05-38.56) | 4.01% (3.71-4.32) | 20.57% (19.94-21.20) | 0.89% (0.74-1.04) | 0.81% (0.67-0.95) | 5.06% (4.72-5.40) | 4.90% (4.56-5.23) |
| Tehran | 11.11% (10.88-11.35) | 30.71% (30.37-31.05) | 6.53% (6.35-6.71) | 43.72% (43.36-44.09) | 24.83% (24.51-25.15) | 1.86% (1.77-1.96) | 41.83% (41.47-42.19) | 9.79% (9.57-10.01) | 33.35% (33.00-33.70) | 1.65% (1.55-1.74) | 5.64% (5.47-5.81) | 4.76% (4.60-4.92) | 9.53% (9.32-9.75) |
| Yazd | 11.91% (11.35-12.46) | 31.77% (30.97-32.56) | 7.19% (6.75-7.63) | 44.45% (43.60-45.30) | 28.85% (28.08-29.63) | 7.43% (6.99-7.88) | 47.73% (46.87-48.58) | 8.66% (8.18-9.14) | 27.92% (27.16-28.69) | 1.10% (0.92-1.27) | 4.22% (3.88-4.57) | 1.97% (1.73-2.21) | 12.42% (11.86-12.99) |
| Zanjan | 25.11% (24.41-25.81) | 71.99% (71.27-72.72) | 8.86% (8.41-9.32) | 71.82% (71.09-72.55) | 34.44% (33.68-35.21) | 3.47% (3.17-3.77) | 58.14% (57.34-58.94) | 17.26% (16.65-17.87) | 55.83% (55.03-56.64) | 11.72% (11.20-12.23) | 15.46% (14.87-16.04) | 11.67% (11.15-12.19) | 30.75% (30.00-31.50) |

**Supplementary Table 2.** Associations between main medication groups and median hospitalization stay.

| **Medication groups** | **Overall hospital stay** | | **ICU stay** | |
| --- | --- | --- | --- | --- |
|  | **MR** | **Adjusted MR^*^** | **MR** | **Adjusted MR^*^** |
| Antithrombotics | 1.58 (1.58-1.59) | 0.94 (0.94-0.95) | 1.08 (1.07-1.08) | 0.97 (0.97-0.98) |
| Antibiotics | 1.56 (1.55-1.56) | 1.12 (1.12-1.13) | 1.11 (1.11-1.11) | 1.05 (1.05-1.05) |
| Corticosteroids | 1.45 (1.44-1.45) | 1.14 (1.14-1.15) | 1.04 (1.04-1.04) | 0.99 (0.99-0.99) |
| Gastrointestinal | 1.48 (1.47-1.48) | 0.99 (0.99-0.99) | 1.09 (1.08-1.09) | 1.03 (1.03-1.03) |
| Antivirals | 1.23 (1.23-1.24) | 1.25 (1.24-1.25) | 0.99 (0.99-0.99) | 0.99 (0.99-1.0) |
| Inhale | 1.63 (1.62-1.64) | 1.13 (1.12-1.13) | 1.1 (1.1-1.1) | 1.03 (1.03-1.04) |
| Diuretics | 2.27 (2.26-2.28) | 1.6 (1.59-1.61) | 1.72 (1.71-1.73) | 1.5 (1.5-1.51) |
| Antidiabetics | 1.88 (1.87-1.89) | 1.28 (1.27-1.28) | 1.18 (1.17-1.18) | 1.09 (1.08-1.09) |
| Immunostimulants | 1.54 (1.53-1.55) | 1.15 (1.15-1.16) | 1.04 (1.04-1.05) | 0.99 (0.99-1.0) |
| ACE inhibitor/ARB | 1.67 (1.67-1.68) | 1.07 (1.07-1.08) | 1.23 (1.22-1.23) | 1.04 (1.04-1.05) |

*MR: median ratio; ICU: intensive care unit. ^*^Adjustments were done with sex, age, the month of admission, province, insurance fund type, admission type, physician specialty, other main groups of medications + Atorvastatin, Vitamin D, Hydroxychloroquine, Colchicine, Nitroglycerin*

**Supplementary Table 3.** Associations between each individual medication and median hospitalization stay.

| **Medication** | **Overall hospital stay** | | **ICU stay** | |
| --- | --- | --- | --- | --- |
|  | **MR** | **Adjusted MR^*^** | **MR** | **Adjusted MR^*^** |
| Heparin | 1.53 (1.53-1.54) | 1.0 (1.0-1.01) | 1.1 (1.1-1.11) | 1.01 (1.01-1.02) |
| Enoxaparin | 1.4 (1.39-1.4) | 1.03 (1.03-1.03) | 1.07 (1.07-1.07) | 1.01 (1.01-1.02) |
| Aspirin | 1.55 (1.55-1.56) | 0.98 (0.98-0.98) | 1.16 (1.15-1.16) | 0.98 (0.98-0.98) |
| Clopidogrel | 1.55 (1.54-1.57) | 0.98 (0.97-0.99) | 1.51 (1.5-1.52) | 1.07 (1.06-1.08) |
| Warfarin | 2.29 (2.24-2.35) | 1.22 (1.2-1.24) | 1.57 (1.54-1.6) | 1.03 (1.02-1.05) |
| Ceftriaxone | 1.31 (1.3-1.31) | 1.0 (0.99-1.0) | 1.03 (1.03-1.04) | 0.98 (0.97-0.98) |
| Azithromycin | 1.32 (1.31-1.32) | 1.01 (1.01-1.02) | 1.01 (1.01-1.02) | 0.96 (0.96-0.96) |
| Vancomycin | 2.67 (2.65-2.68) | 1.35 (1.34-1.36) | 1.94 (1.93-1.95) | 1.26 (1.25-1.27) |
| Meropenem | 2.61 (2.59-2.63) | 1.31 (1.31-1.32) | 1.9 (1.89-1.92) | 1.24 (1.23-1.24) |
| Cefepime | 1.87 (1.85-1.89) | 1.18 (1.17-1.19) | 1.22 (1.21-1.23) | 1.06 (1.05-1.07) |
| Ceftazidime | 1.66 (1.64-1.67) | 1.09 (1.08-1.1) | 1.14 (1.14-1.15) | 1.02 (1.01-1.02) |
| Imipenem | 2.5 (2.48-2.53) | 1.28 (1.27-1.29) | 1.67 (1.65-1.68) | 1.18 (1.17-1.18) |
| Cefotaxime | 1.3 (1.27-1.32) | 1.26 (1.24-1.28) | 1.12 (1.11-1.14) | 1.08 (1.07-1.09) |
| Levofloxacin | 1.62 (1.58-1.66) | 1.17 (1.14-1.19) | 1.23 (1.21-1.25) | 1.01 (1.0-1.03) |
| ceftizoxime | 1.25 (1.22-1.29) | 1.06 (1.04-1.08) | 1.05 (1.03-1.06) | 1.03 (1.02-1.05) |
| Dexamethasone | 1.36 (1.35-1.36) | 1.09 (1.08-1.09) | 1.02 (1.02-1.02) | 0.98 (0.98-0.98) |
| Methylprednisolone | 1.95 (1.93-1.96) | 1.22 (1.21-1.23) | 1.25 (1.25-1.26) | 1.1 (1.09-1.1) |
| Hydrocortisone | 1.75 (1.74-1.77) | 1.1 (1.09-1.11) | 1.35 (1.34-1.36) | 1.09 (1.09-1.1) |
| Prednisolone | 2.13 (2.11-2.16) | 1.26 (1.25-1.27) | 1.19 (1.18-1.2) | 1.03 (1.03-1.04) |
| Betamethasone (IM/IV) | 1.46 (1.42-1.5) | 1.16 (1.14-1.18) | 1.1 (1.09-1.12) | 1.04 (1.02-1.05) |
| Pantoprazole | 1.46 (1.46-1.47) | 0.98 (0.98-0.98) | 1.16 (1.16-1.16) | 1.02 (1.02-1.02) |
| Famotidine | 1.37 (1.37-1.38) | 0.96 (0.95-0.96) | 1.01 (1.01-1.01) | 0.97 (0.97-0.98) |
| Omeprazole | 1.59 (1.55-1.63) | 1.17 (1.14-1.19) | 1.04 (1.03-1.06) | 0.98 (0.97-0.99) |
| Remdesivir | 1.18 (1.18-1.19) | 1.24 (1.24-1.25) | 0.98 (0.98-0.98) | 1.0 (0.99-1.0) |
| Lopinavir/ritonavir | 1.51 (1.49-1.53) | 1.15 (1.14-1.17) | 1.04 (1.03-1.05) | 1.0 (0.99-1.01) |
| Favipiravir | 1.33 (1.31-1.34) | 1.02 (1.01-1.03) | 1.01 (1.0-1.01) | 0.98 (0.97-0.99) |
| Acyclovir | 2.59 (2.53-2.66) | 1.32 (1.29-1.34) | 1.44 (1.41-1.47) | 1.08 (1.07-1.1) |
| Atorvastatin | 1.58 (1.57-1.58) | 1.0 (1.0-1.0) | 1.13 (1.13-1.13) | 0.97 (0.97-0.98) |
| Fluticasone/salmeterol | 1.6 (1.59-1.61) | 1.03 (1.02-1.03) | 1.08 (1.07-1.08) | 0.98 (0.98-0.99) |
| Salbutamol | 1.56 (1.55-1.57) | 1.03 (1.02-1.04) | 1.16 (1.15-1.16) | 1.02 (1.02-1.02) |
| Atrovent | 1.76 (1.74-1.77) | 1.07 (1.06-1.08) | 1.21 (1.2-1.21) | 1.03 (1.02-1.03) |
| Furosemide | 2.31 (2.3-2.33) | 1.29 (1.28-1.29) | 1.79 (1.78-1.8) | 1.23 (1.22-1.23) |
| Spironolactone | 2.21 (2.19-2.24) | 1.13 (1.12-1.14) | 2.1 (2.08-2.13) | 1.15 (1.14-1.17) |
| Hydrochlorothiazide | 1.92 (1.88-1.97) | 1.12 (1.1-1.13) | 1.35 (1.32-1.37) | 1.06 (1.05-1.08) |
| Insulins | 1.95 (1.94-1.96) | 1.19 (1.18-1.19) | 1.21 (1.21-1.22) | 1.06 (1.06-1.07) |
| Metformin | 1.56 (1.55-1.58) | 1.04 (1.03-1.04) | 1.02 (1.02-1.03) | 0.95 (0.94-0.95) |
| Losartan | 1.69 (1.67-1.7) | 1.03 (1.02-1.03) | 1.21 (1.2-1.21) | 1.01 (1.01-1.02) |
| Captopril | 1.59 (1.58-1.61) | 1.03 (1.02-1.03) | 1.29 (1.28-1.3) | 1.04 (1.03-1.04) |
| Valsartan | 1.76 (1.74-1.79) | 1.01 (1.0-1.02) | 1.34 (1.32-1.35) | 1.02 (1.01-1.03) |
| Amlodipine | 2.08 (2.06-2.09) | 1.18 (1.17-1.19) | 1.53 (1.52-1.54) | 1.16 (1.15-1.17) |
| Metoprolol | 1.86 (1.84-1.87) | 1.09 (1.08-1.1) | 1.43 (1.42-1.44) | 1.09 (1.08-1.09) |
| Nitroglycerin | 1.73 (1.72-1.74) | 1.01 (1.0-1.02) | 1.52 (1.51-1.53) | 1.09 (1.08-1.1) |
| Norepinephrine | 5.02 (4.95-5.09) | 2.08 (2.06-2.11) | 9.75 (9.65-9.86) | 5.09 (5.03-5.14) |
| Digoxin | 2.57 (2.53-2.61) | 1.16 (1.14-1.18) | 3.68 (3.62-3.73) | 1.58 (1.56-1.6) |
| Interferon beta-1a | 1.49 (1.48-1.5) | 1.15 (1.09-1.21) | 1.03 (1.03-1.04) | 0.97 (0.94-1.01) |
| Interferon beta-1b | 1.68 (1.64-1.72) | 1.23 (1.17-1.29) | 1.06 (1.05-1.08) | 1.02 (0.98-1.06) |
| Filgrastim (GCSF) | 2.96 (2.85-3.07) | 1.45 (1.37-1.53) | 1.38 (1.34-1.41) | 0.95 (0.91-0.99) |
| Vitamin D3 | 1.44 (1.43-1.45) | 1.03 (1.02-1.03) | 1.06 (1.06-1.06) | 1.02 (1.01-1.02) |
| Vitamin C | 1.22 (1.2-1.23) | 1.09 (1.08-1.11) | 1.11 (1.1-1.12) | 1.05 (1.04-1.06) |
| Hydroxychloroquine | 1.38 (1.37-1.4) | 1.05 (1.04-1.06) | 1.02 (1.02-1.03) | 0.95 (0.95-0.96) |
| Colchicine | 1.85 (1.83-1.87) | 1.13 (1.12-1.14) | 1.18 (1.18-1.19) | 1.06 (1.06-1.07) |

*^*^ Adjustments were done with sex, age, the month of admission, province, insurance fund type, admission type, physician specialty, and other drugs in this table.*

**Supplementary Table 4.** Associations between main medication groups and probability of mortality.

| **Medication groups** | **All cases** | | **ICU admitted cases** | |
| --- | --- | --- | --- | --- |
|  | **OR** | **Adjusted OR^*^** | **OR** | **Adjusted OR^*^** |
| Antithrombotics | 1.64 (1.61-1.66) | 0.74 (0.73-0.76) | 1.06 (1.04-1.09) | 0.66 (0.64-0.69) |
| Antibiotics | 2.39 (2.36-2.43) | 1.71 (1.68-1.74) | 1.49 (1.46-1.52) | 1.6 (1.55-1.65) |
| Corticosteroids | 1.25 (1.23-1.27) | 0.97 (0.95-0.99) | 1.39 (1.36-1.42) | 1.31 (1.26-1.35) |
| Gastrointestinal | 1.91 (1.89-1.94) | 1.29 (1.26-1.31) | 1.29 (1.27-1.32) | 1.22 (1.18-1.26) |
| Antivirals | 0.69 (0.68-0.7) | 0.82 (0.8-0.83) | 1.1 (1.07-1.12) | 1.02 (0.99-1.05) |
| Inhalers | 1.63 (1.6-1.66) | 1.04 (1.02-1.07) | 1.09 (1.06-1.12) | 0.88 (0.85-0.91) |
| Diuretics | 4.8 (4.73-4.88) | 2.99 (2.93-3.05) | 1.49 (1.46-1.52) | 1.55 (1.51-1.59) |
| Antidiabetics | 2.14 (2.11-2.18) | 1.32 (1.29-1.34) | 1.26 (1.23-1.29) | 1.07 (1.03-1.1) |
| Immunostimulants | 1.46 (1.43-1.49) | 1.01 (0.98-1.03) | 1.33 (1.29-1.38) | 1.14 (1.09-1.19) |
| ACE inhibitor/ARB | 1.99 (1.96-2.03) | 0.79 (0.77-0.8) | 0.89 (0.87-0.91) | 0.68 (0.66-0.7) |

*^*^ Adjustments were done with sex, age, the month of admission, province, insurance fund type, admission type, physician specialty, other main groups of medications + Atorvastatin, Vitamin D, Hydroxychloroquine, Colchicine, Nitroglycerin*

**Supplementary Table 5.** Associations between each medication and mortality.

| **Medication** | **All cases** | | **ICU admitted cases** | |
| --- | --- | --- | --- | --- |
|  | **OR** | **Adjusted OR^*^** | **OR** | **Adjusted OR^*^** |
| Heparin | 2.04 (2.01-2.06) | 1.11 (1.08-1.13) | 1.32 (1.3-1.35) | 0.98 (0.94-1.01) |
| Enoxaparin | 1.29 (1.27-1.31) | 0.83 (0.81-0.85) | 0.98 (0.95-1.0) | 0.8 (0.78-0.83) |
| Aspirin | 2.01 (1.98-2.04) | 0.84 (0.82-0.86) | 0.99 (0.97-1.02) | 0.85 (0.82-0.88) |
| Clopidogrel | 2.42 (2.35-2.48) | 0.95 (0.91-0.99) | 0.71 (0.69-0.74) | 0.76 (0.73-0.8) |
| Warfarin | 2.22 (2.08-2.37) | 0.61 (0.56-0.67) | 0.76 (0.7-0.82) | 0.56 (0.5-0.62) |
| Ceftriaxone | 1.34 (1.32-1.36) | 0.84 (0.82-0.86) | 1.07 (1.04-1.09) | 0.92 (0.89-0.95) |
| Azithromycin | 1.14 (1.11-1.16) | 0.68 (0.66-0.7) | 0.92 (0.88-0.95) | 0.81 (0.77-0.85) |
| Vancomycin | 6.11 (6.01-6.2) | 2.15 (2.09-2.2) | 2.11 (2.06-2.16) | 1.48 (1.43-1.53) |
| Meropenem | 5.87 (5.77-5.97) | 1.86 (1.81-1.9) | 2.14 (2.09-2.19) | 1.45 (1.41-1.5) |
| Cefepime | 2.71 (2.64-2.78) | 1.36 (1.31-1.41) | 1.66 (1.6-1.72) | 1.26 (1.2-1.32) |
| Ceftazidime | 2.01 (1.96-2.07) | 1.11 (1.07-1.15) | 1.26 (1.21-1.31) | 1.03 (0.98-1.08) |
| Imipenem | 4.14 (4.04-4.26) | 1.68 (1.62-1.74) | 1.76 (1.69-1.82) | 1.37 (1.3-1.43) |
| Cefotaxime | 0.75 (0.69-0.82) | 1.03 (0.93-1.15) | 0.4 (0.36-0.44) | 0.85 (0.75-0.96) |
| Levofloxacin | 1.19 (1.08-1.31) | 0.93 (0.83-1.03) | 0.78 (0.67-0.89) | 0.93 (0.82-1.05) |
| ceftizoxime | 1.05 (1.03-1.06) | 1.01 (0.89-1.13) | 1.29 (1.26-1.31) | 0.8 (0.68-0.94) |
| Dexamethasone | 2.69 (2.65-2.74) | 0.82 (0.8-0.84) | 1.57 (1.53-1.61) | 1.03 (0.99-1.06) |
| Methylprednisolone | 3.67 (3.6-3.75) | 2.06 (2.01-2.11) | 1.75 (1.7-1.8) | 1.56 (1.51-1.62) |
| Hydrocortisone | 1.06 (1.01-1.1) | 1.65 (1.6-1.69) | 0.49 (0.47-0.51) | 1.31 (1.26-1.36) |
| Prednisolone | 1.21 (1.11-1.31) | 0.39 (0.37-0.42) | 0.86 (0.77-0.96) | 0.34 (0.32-0.36) |
| Betamethasone (IM/IV) | 2.54 (2.51-2.58) | 0.84 (0.76-0.94) | 1.43 (1.4-1.46) | 0.91 (0.8-1.04) |
| Pantoprazole | 1.12 (1.1-1.14) | 1.22 (1.2-1.25) | 1.13 (1.11-1.16) | 1.09 (1.06-1.12) |
| Famotidine | 0.93 (0.84-1.03) | 0.81 (0.79-0.83) | 0.68 (0.6-0.78) | 0.93 (0.9-0.96) |
| Omeprazole | 0.63 (0.62-0.64) | 0.67 (0.59-0.75) | 1.07 (1.05-1.1) | 0.7 (0.6-0.82) |
| Remdesivir | 1.58 (1.51-1.65) | 0.78 (0.76-0.8) | 1.34 (1.25-1.43) | 1.02 (0.98-1.05) |
| Lopinavir/ritonavir | 1.11 (1.06-1.17) | 1.03 (0.98-1.09) | 1.04 (0.96-1.13) | 1.12 (1.03-1.22) |
| Favipiravir | 2.91 (2.74-3.1) | 0.86 (0.81-0.92) | 1.13 (1.04-1.23) | 0.94 (0.85-1.03) |
| Acyclovir | 1.96 (1.93-1.98) | 1.24 (1.14-1.35) | 1.06 (1.04-1.09) | 0.99 (0.89-1.1) |
| Atorvastatin | 1.46 (1.43-1.49) | 0.84 (0.82-0.86) | 1.05 (1.02-1.08) | 0.84 (0.81-0.87) |
| Fluticasone/salmeterol | 2.1 (2.06-2.15) | 0.85 (0.83-0.88) | 1.23 (1.2-1.27) | 0.82 (0.79-0.86) |
| Salbutamol | 2.19 (2.13-2.25) | 1.12 (1.09-1.16) | 1.13 (1.09-1.18) | 1.03 (0.99-1.07) |
| Atrovent | 5.06 (4.98-5.14) | 0.98 (0.94-1.02) | 1.58 (1.55-1.62) | 0.9 (0.86-0.95) |
| Furosemide | 2.9 (2.81-3.0) | 2.06 (2.01-2.11) | 0.78 (0.75-0.82) | 1.39 (1.34-1.43) |
| Spironolactone | 1.84 (1.73-1.96) | 0.68 (0.65-0.72) | 0.83 (0.77-0.9) | 0.65 (0.61-0.68) |
| Hydrochlorothiazide | 2.38 (2.33-2.42) | 0.86 (0.8-0.94) | 1.32 (1.29-1.36) | 0.86 (0.78-0.94) |
| Insulins | 0.78 (0.74-0.82) | 1.16 (1.13-1.19) | 0.63 (0.59-0.67) | 0.99 (0.95-1.02) |
| Metformin | 1.9 (1.87-1.94) | 0.56 (0.53-0.6) | 0.96 (0.93-0.98) | 0.65 (0.6-0.7) |
| Losartan | 1.97 (1.91-2.02) | 0.78 (0.76-0.8) | 0.79 (0.76-0.82) | 0.76 (0.73-0.78) |
| Captopril | 2.05 (1.97-2.14) | 0.88 (0.85-0.91) | 0.89 (0.84-0.94) | 0.81 (0.77-0.85) |
| Valsartan | 2.98 (2.91-3.04) | 0.8 (0.76-0.85) | 1.19 (1.16-1.23) | 0.79 (0.74-0.85) |
| Amlodipine | 2.79 (2.72-2.86) | 1.14 (1.11-1.18) | 1.13 (1.1-1.17) | 1.0 (0.96-1.04) |
| Metoprolol | 3.0 (2.94-3.06) | 1.18 (1.14-1.22) | 0.92 (0.89-0.94) | 1.04 (1.0-1.08) |
| Nitroglycerin | 30.54 (29.77-31.34) | 1.15 (1.12-1.19) | 8.76 (8.46-9.09) | 0.89 (0.86-0.93) |
| Norepinephrine | 7.6 (7.36-7.86) | 16.83 (16.3-17.37) | 2.0 (1.91-2.08) | 8.7 (8.34-9.07) |
| Digoxin | 1.38 (1.34-1.41) | 2.2 (2.11-2.31) | 1.3 (1.25-1.35) | 1.53 (1.45-1.62) |
| Interferon beta-1a | 1.13 (1.03-1.23) | 0.89 (0.86-0.92) | 0.97 (0.85-1.09) | 1.05 (1.0-1.1) |
| Interferon beta-1b | 4.13 (3.8-4.48) | 0.96 (0.86-1.08) | 2.29 (2.02-2.6) | 0.76 (0.65-0.89) |
| Filgrastim (GCSF) | 1.32 (1.29-1.35) | 1.63 (1.46-1.81) | 1.1 (1.07-1.13) | 1.97 (1.69-2.3) |
| Vitamin D3 | 1.27 (1.2-1.34) | 0.9 (0.87-0.92) | 0.91 (0.85-0.98) | 0.88 (0.85-0.92) |
| Vitamin C | 1.37 (1.32-1.42) | 1.06 (0.98-1.15) | 1.18 (1.11-1.24) | 0.98 (0.89-1.08) |
| Hydroxychloroquine | 1.74 (1.68-1.8) | 0.74 (0.7-0.77) | 1.04 (0.99-1.09) | 0.98 (0.92-1.06) |
| Colchicine | 2.04 (2.01-2.06) | 1.13 (1.08-1.19) | 1.32 (1.3-1.35) | 0.94 (0.89-1.0) |

*^*^ Adjustments were done with sex, age, the month of admission, province, insurance fund type, admission type, physician specialty, and other drugs in this table.*
