# Supplementary material for: Drug prescription patterns and their association with mortality and hospitalization duration in COVID-19 patients: insights from big data
Source: Front Public Health. 2023 Dec 18;11:1280434. doi: 10.3389/fpubh.2023.1280434 (PMC10758044; doi:10.3389/fpubh.2023.1280434)

Supplementary Figure 1. Percentage of medication prescriptions among cases with COVID-19 for different main groups of drugs based on age and sex.

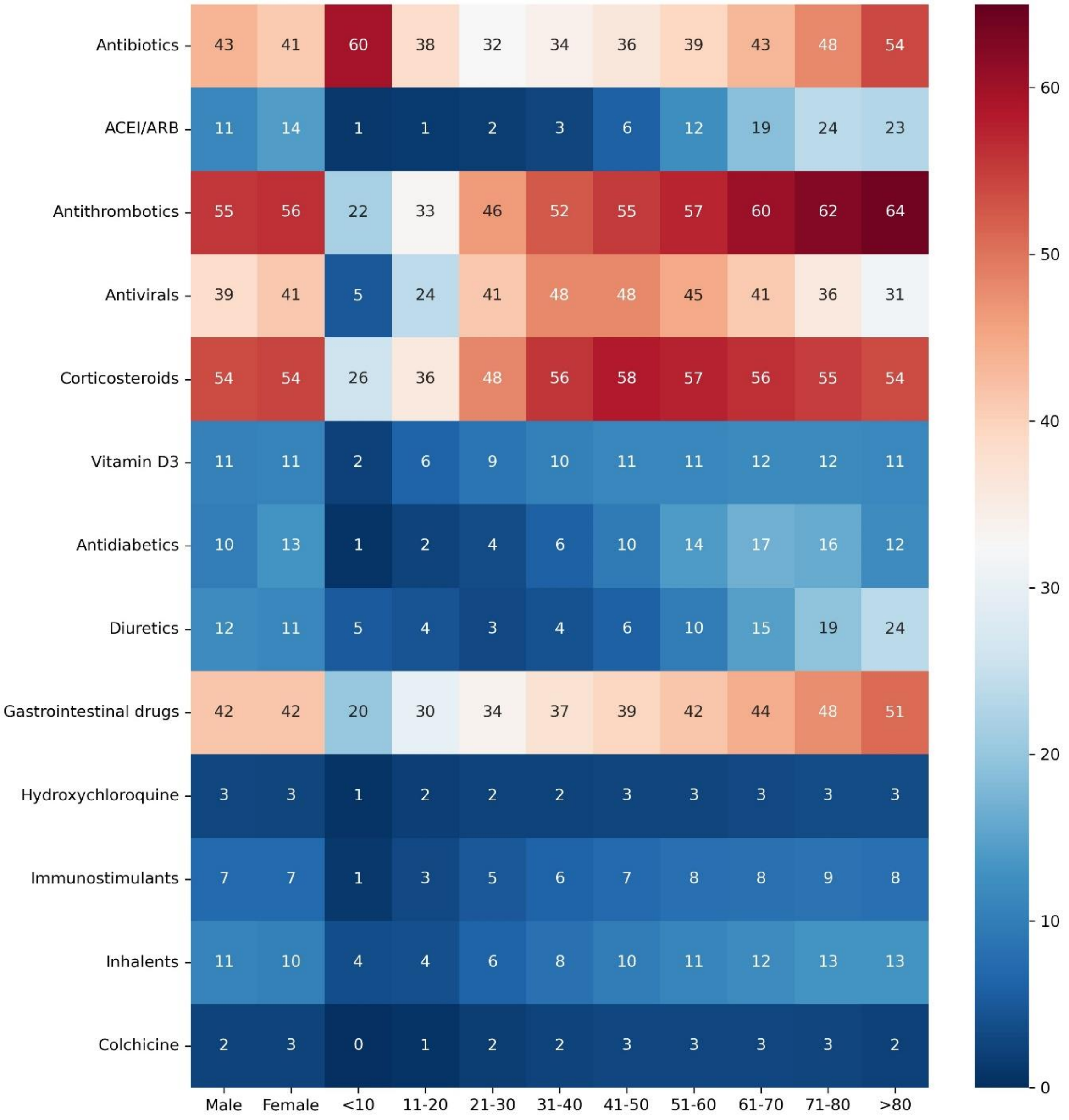

Supplementary Figure 2. Medication prescription percentage among cases with COVID-19 for different main groups of drugs based on different specialties.

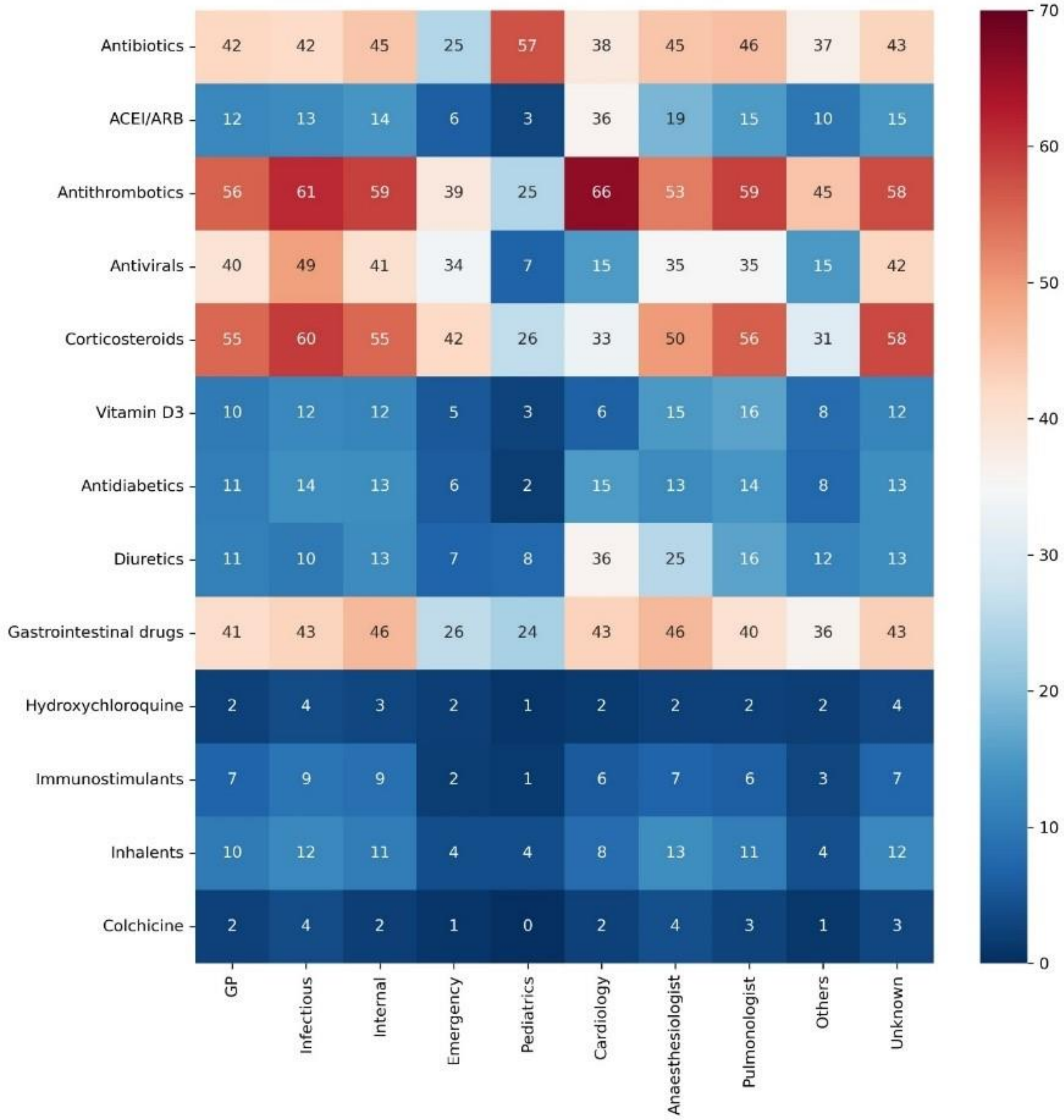

Supplement: Supplementary file 2 [file Data_Sheet_2.pdf]
